# High-Throughput Screening to Predict Chemical-Assay Interference

**DOI:** 10.1038/s41598-020-60747-3

**Published:** 2020-03-04

**Authors:** Alexandre Borrel, Ruili Huang, Srilatha Sakamuru, Menghang Xia, Anton Simeonov, Kamel Mansouri, Keith A. Houck, Richard S. Judson, Nicole C. Kleinstreuer

**Affiliations:** 1 NIH/NIEHS/DIR/BCBB, RTP, NC United States; 2 NIH/NCATS, Bethesda, MD United States; 3 Integrated Laboratory Systems, RTP, NC United States; 4 EPA/ORD/CCTE, RTP, NC United States; 5 NIH/NIEHS/DNTP/NICEATM, RTP, NC United States

**Keywords:** Bioinformatics, High-throughput screening, Screening, Computational platforms and environments

## Abstract

The U.S. federal consortium on toxicology in the 21^st^ century (Tox21) produces quantitative, high-throughput screening (HTS) data on thousands of chemicals across a wide range of assays covering critical biological targets and cellular pathways. Many of these assays, and those used in other *in vitro* screening programs, rely on luciferase and fluorescence-based readouts that can be susceptible to signal interference by certain chemical structures resulting in false positive outcomes. Included in the Tox21 portfolio are assays specifically designed to measure interference in the form of luciferase inhibition and autofluorescence via multiple wavelengths (red, blue, and green) and under various conditions (cell-free and cell-based, two cell types). Out of 8,305 chemicals tested in the Tox21 interference assays, percent actives ranged from 0.5% (red autofluorescence) to 9.9% (luciferase inhibition). Self-organizing maps and hierarchical clustering were used to relate chemical structural clusters to interference activity profiles. Multiple machine learning algorithms were applied to predict assay interference based on molecular descriptors and chemical properties. The best performing predictive models (accuracies of ~80%) have been included in a web-based tool called InterPred that will allow users to predict the likelihood of assay interference for any new chemical structure and thus increase confidence in HTS data by decreasing false positive testing results.

## Introduction

Chemical hazard assessment testing in the twenty-first century has evolved to encompass large high-throughput screening (HTS) research programs, designed to produce quantitative data on the activity of thousands of chemicals across hundreds of biological targets and pathways, a strategy that has long been used in drug discovery. Such efforts, exemplified by the federal Tox21 research consortium^1,2^, are intended to facilitate rapid chemical hazard screening, predictive computational toxicology using machine learning and artificial intelligence techniques, and human-relevant systems biology models that provide mechanistic insight into chemical toxicity (e.g.^3^). The Tox21 program and other such HTS initiatives rely upon an array of biological assays, i.e. analytical measurement procedures defined by a set of reagents that produce a detectable signal, allowing a biological process to be quantified^4^. Many HTS platforms use cell-based assays measuring processes such as cell growth/death, receptor binding, or protein expression while others are cell-free assays that characterize biochemical activity. Both formats use a variety of detection technologies including fluorescence and luminescence readouts. Fluorescence-based assays are one of the leading technologies in terms of widespread application reported in PubChem^5,6^. Indeed, the routinely used fluorescence intensity-based format allows for optimization of speed, accuracy, reproducibility and sensitivity of assays^7^. Luminescence is frequently used as a readout from luciferase-based reporter gene assays and provides high sensitivity due to lack of background activity in mammalian cell lines.

The mechanisms of signal generation from these two common assay formats differ: (1) luciferase expression level is quantitated by the luminescence produced by the luciferase-catalyzed oxidation of added luciferin substrate and (2) fluorescence intensity is measured by excitation at a wavelength matching the fluorescent substrate coupled to quantitation of the wavelength emitted by the excited fluorophore^8^. Each are subject to different interferent chemicals, i.e. chemicals that modulate the signal intensity without any biological action. There are two main mechanisms by which a compound can directly interfere with a fluorescent assay: quenching, i.e. chemicals absorb light directly, and autofluorescence, i.e. chemicals emit light that overlaps the fluorophore spectrum^4^. With respect to luciferase, chemicals can interfere by inhibiting luciferase enzymatic activity and possibly by direct oxidation of the luciferin substrate^9^. These phenomena are not isolated to a few chemicals, rather more than 5% of PubChem chemical libraries (from over 70,000 tested samples), may have autofluorescence properties^10^, and 12% of active chemicals from the NIH Molecular Libraries Small Molecule Repository give paradoxical luminescence changes^11^. These phenomena also impact many drug discovery campaigns and have been reported and documented for various targets and methods, e.g. kinases or proteases^12^.

Considering the importance of HTS platforms in drug discovery and chemical toxicity screening, and the potential impact of false signals derived from these two major interference mechanisms, standardized *in silico* tools are needed to predict and limit interferent compounds being misinterpreted in fluorescent assay technologies. Adequately trained interference prediction models based on chemical structure will help avoid artefacts and false signals, conserve time and resources, and allow for optimized screening approaches. Correlations between chemical structure and fluorescence/interference have been studied previously, and most approaches have relied upon identifying chemicals with particular substructures as problematic, e.g. thiol or quinone substructures^13^, or rule-based alerts on ring structures/properties^14^. A popular example in drug discovery to guide HTS library conception is the Pan Assay Interference Compounds (PAINS) approach, where a set of chemicals/substructures were identified as interferent due to non-selective activity^15^. However, the success of existing methods to define interferents is still limited due to the complexity and multifactorial nature of the platforms, such as cell culture, range of absorbance in the light spectrum, detection technology, fluorophore used, etc. which necessitate specific studies to directly measure chemical-assay interference rather than inferring interference patterns using datasets measuring biological activity.

The Tox21 library includes 10K chemicals (8,305 unique structures) tested in a variety of assay technologies, many of which rely upon fluorescence and luciferase-based readouts to indicate biological activity^16,17^. Here we present the results from a subset of Tox21 assays specifically designed to measure interference in the form of luciferase inhibition and autofluorescence. A single assay was used to measure luciferase inhibition in a cell-free format. An additional 12 assay endpoints were derived by screening two cell types (HEK-293 and HepG2) at three different fluorescent wavelengths (red, blue, green) under cell culture conditions with and without cells. The Tox21 library, focused on environmentally relevant and potentially toxic chemicals, was screened in these assays using a quantitative HTS protocol^18^, representing one of the largest screening efforts for chemical-assay interference to date. Using a set of 1D and 2D molecular descriptors covering both physicochemical and topological chemical properties, Self-Organizing Maps (SOM) and hierarchical clustering were applied to characterize interferent chemicals. Machine learning approaches were leveraged to build statistical quantitative structure–activity relationships (QSAR) models that use selected molecular descriptors to predict the probability of a chemical to interfere with fluorescent intensity or luciferase assays; these open-source models are freely accessible for new chemical prediction via a web-based interface called InterPred (https://sandbox.ntp.niehs.nih.gov/interferences/).

## Material and Methods

### Assays

Three assay platforms were applied to analyze luciferase and fluorescence interference patterns using the Tox21 chemical screening library. The raw data are freely available on the NCATS Tox21 browser (https://tripod.nih.gov/tox21/assays/) under the names “tox21-luc-biochem-p1” for the luciferase inhibition assay, and “tox21-spec-hepg2-p1” and “tox21-spec-hek293-p1” for autofluorescence assays using HepG2 and HEK-293 cell cultures, respectively, measuring red, green and blue wavelengths using cell-based and cell-free culture-medium-only conditions. The Tox21 chemical library (8,305 unique substances) was screened in triplicate concentration response in all assays, with concurrent cytotoxicity measurements where applicable.

#### Luciferase assays

##### Reagents

The substrate D-Luciferin and the enzyme firefly-Luciferase were purchased from Sigma-Aldrich (St. Louis, MO). The positive control compound (PTC-124) was purchased from Santa Cruz Biotechnology, Inc. (Dallas, TX).

##### Luciferase (biochemical) qHTS assay

Three µL of the substrate (mixture containing 50 mM Tris-acetate pH 7.6, 13.3 mM magnesium acetate, 0.01 mM D-luciferin, 0.01 mM ATP, 0.01% Tween, 0.05% BSA and distilled H_2_O) was dispensed into medium-binding white/solid 1,536-well plates (Greiner Bio-One North America Inc., Monroe, NC) using a Flying Reagent Dispenser, FRD (Aurora Discovery, Carlsbad, CA). Then 23 nL of the test compounds (into 5–48 columns), PTC-124 and/or DMSO (into 1–4 columns) were transferred to the assay plates using a Pintool station (Wako, San Diego, CA). The positive control plate format is, column-1: sixteen-concentration two-fold titration of PTC-124 from 0.035 nM to 1.15 µM with duplicate points; columns-2 & 3 for the top half portions: 0.58 and 1.15 µM PTC-124 respectively and DMSO in the rest. Compound addition was followed by the addition of 1 μl of 10 nM enzyme (mixture containing 50 mM Tris-acetate, 0.04 µM firefly-Luciferase and distilled H_2_O) by using an FRD to all the wells of assay plate except in the 4th column which received the buffer instead. After 5 min incubation at room temperate, luminescence intensity was measured by a Viewlux plate reader (Perkin Elmer, Waltham, MA). Data were expressed as relative luminescence units. Each test compound was measured at 15 concentrations ranging from 1.5 nM to 115 µM and in triplicate.

##### Data analysis

For primary data analysis, raw plate reads for each titration point were first normalized relative to DMSO-only wells that received firefly-Luciferase (basal, 0%) and PTC-124 control wells that received firefly-Luciferase (0.58 µM, −100%) and then corrected by applying a pattern correction algorithm using compound-free control plates (DMSO plates). Concentration-response titration points for each compound replicate were fitted to the Hill equation and concentrations of half-maximal inhibition activity (IC_50_) and maximal response (efficacy) values were calculated^19^; the mean of the IC_50_s was used as the representative value. Concentration response curves were designated as class 1–4 based on the quality of the fit, the number of points above background, and the response efficacy, as previously described (summarized in Supporting Information Table S1)^18,20^.

#### Autofluorescence assays

##### Cell culture

HepG2 and HEK-293 cells were purchased from American Type Culture Collection (ATCC, Manassas, VA). HepG2 cells were cultured in Eagle’s Minimum Essential Medium (EMEM, ATCC) and HEK-293 cells in Dulbecco’s Modified Eagle’s Medium (DMEM, Life Technologies Corporation, Carlsbad, CA), and both supplemented with 10% Hyclone’s Fetal Bovine Serum (ThermoFisher Scientific, Waltham, MA), and 100 U/ml penicillin-100 µg/ml streptomycin (Life Technologies Corporation). Cells were cultured and maintained at 37 °C under a humidified atmosphere and 5% CO_2_.

##### Auto-fluorescence qHTS assays in cells or culture media

HepG2 and HEK-293 cells were dispensed at 2,000/well in 5 µL of the culture medium into 1,536-well black/clear plates using a Multidrop™ Combi Reagent dispenser (ThermoFisher Scientific). In parallel, 5 µL/well of culture medium only were dispensed into 1,536-well black/clear plates. For cell attachment to plate well bottom, HepG2 cells or HepG2 culture medium-alone assay plates were incubated overnight at 37 °C, whereas HEK-293 cells/HEK-293 culture medium-alone assay plates were incubated for 5 hr at 37 °C. After respective incubation times, 23 nL of the compounds dissolved in DMSO, positive controls (fluorescein-green, triamterene-blue, and rose bengal-red) were transferred to the assay plates using Pintool station (Wako, San Diego, CA). Then the assay plates were incubated at 37 °C for 1 hr (HepG2 cells/HepG2 culture medium-alone plates) and 16 hr (HEK-293 cells/HEK-293 culture medium-alone plates). The fluorescence intensity was measured using an Envision plate reader (Perkin Elmer, Waltham, MA) by using excitation/emission filters at 485/535 nm, 405/460 nm, and 540/590 nm for green, blue and red channels respectively. Data were expressed as relative fluorescence units. Each test compound was measured at 5 concentrations ranging from to 29 nM to 92 µM.

##### Data analysis

Analysis of compound concentration-response data was performed as previously described^21^. Briefly, raw plate reads for each titration point were first normalized and then corrected by applying a pattern correction algorithm using compound-free control plates (DMSO plates)^22^. The responses from the positive control compounds were much higher than many of the fluorescent compounds in the library, some by several orders of magnitude. In order to see responses from weaker fluorescent compounds, the data were normalized in two different ways: (1) using the respective positive control compound and (2) using negative controls (DMSO-only wells). When normalizing to the positive control, the positive control response was set to 100% and negative control to 0% as follows:

$${\rm{Activity}}\,( \% )=([{{\rm{V}}}_{{\rm{c}}}-{{\rm{V}}}_{{\rm{DMSO}}}]/[{{\rm{V}}}_{{\rm{pos}}}-{{\rm{V}}}_{{\rm{DMSO}}}])\times 100,$$

where V_c_ denotes the compound well values, V_pos_ denotes the median value of the positive control wells, and V_DMSO_ denotes the median values of the DMSO-only wells. Fluorescein (0.32 µM for HepG2 and 0.6 µM for HEK-293) was used as the positive control for green fluorescence, triamterene (20 µM) for blue fluorescence and rose bengal control (50 µM) for red fluorescence. The following formula was used to normalize data to the negative control:

$${\rm{Activity}}\,( \% )=([{{\rm{V}}}_{{\rm{c}}}-{{\rm{V}}}_{{\rm{DMSO}}}]/[{{\rm{V}}}_{{\rm{DMSO}}}])\times 100$$

Concentration-response titration points for each compound were fitted to the Hill equation and concentrations of half-maximal activity (AC_50_) and maximal response (efficacy) values were calculated^19^. Concentration response curves were designated as class 1–4 based on the quality of the fit, the number of points above background, and the response efficacy, as previously described (summarized in Supporting Information Table S1)^18,20^.

### Active chemical identification

Chemicals were first filtered based on potency by only considering AC_50_ (autofluorescence)/IC_50_ (luciferase inhibition) values below 150 µM, and then based on curve class (i.e. only considered active with curve class 1.x and 2.x for AC_50_ and −1.x and −2.x for IC_50_; see Supporting Information Table S1 for details). To increase the confidence of the measurements, only AC_50_/IC_50_ with efficacy above 30% were considered. Finally, for autofluorescence assays, to avoid indications of generalized cell stress without specific biomolecular interactions, only AC_50_ below the burst cutoff defined in^23^ were considered. Indeed, activity above this limit may result from triggering cell stress pathways, chemical reactivity, physico-chemical disruption of proteins or membranes, or broad low-affinity non-covalent interactions and not from a specific technology interference reaction. This filter was applied to distinguish chemical-structure driven autofluorescence from the increase in signal that has been observed due to cell stress^24^. The distribution of the curve classes for active chemicals is presented in Supporting Information Fig. S1.

### Molecular modeling

Each chemical was encoded using a unique SMILES string format and Chemical Abstract Services Registry Number (CASRN). Data chemical curation and descriptor selection followed the best practices protocol available in^25^. Using the MolVS python library available on (http://molvs.readthedocs.io/en/latest/guide/intro.html) each chemical has been prepared from original SMILES including the following steps: hydrogen removing, sanitization, metal disconnection, stereochemistry process, desolvation, and filtering of salt fragments. Mixtures were not considered and were excluded in an early step. From the Tox21 library (8305 unique substances), 8065 chemicals passed thought the structure curation and were used for clustering and QSAR modeling.

From each curated structure, a set of 677 2D descriptors was computed. A set of 647 descriptors including topological and physicochemical descriptors were computed using RDKit tool kit^26^ implemented in the PyDPI python library^27^, and an additional set of 30 physicochemical descriptors were computed using the OPERA models^28^.

Only informative and non-correlated descriptors were selected for subsequent analyses. Descriptors with a null variance and no discriminant, i.e. the same value for more than 90% of the chemicals, were removed. Pair-wise Person’s correlation coefficient (ρ) was computed for each combination of descriptors, those with ρ > 0.9 were clustered, and one descriptor per cluster was randomly chosen. Molecular descriptors computation was performed in Python 2.7 using standard libraries. Statistical descriptors selection and clustering was performed in R 3.4.4^29^.

### Clustering

Chemicals were characterized using selected descriptors and clustered using Self-Organizing Maps (SOM)^30^ and hierarchical clustering based on a Euclidian distance matrix and Ward linkage^31,32^. SOM was used to identify structural clusters in the entire Tox21 library that were enriched for interference activity, and hierarchical clustering was performed on only the active chemical set for each assay to identify structural features related to potency or interference activity specific to cell types/culture conditions. SOM plots were developed using the R library *kohonen*.

### QSAR classification

#### Machine learning

The QSAR modeling workflow was conducted according to the best practices^33–35^. Classification models to predict active versus inactive chemicals for each of the interference assay endpoints were built using four machine learning approaches: (i) Linear Discriminant Analysis (LDA) based on Fisher’s linear discriminant methods^36^, (ii) Random Forests (RF)^37^, (iii) Support Vector Machine (SVM) with a linear, radial and sigmoid kernel^38^,(iv) Classification and Regression Tree (CART)^39^ and a neural network^40,41^. QSAR models were built using R packages: *pls*, *randomForest*, *rpart*, *e1071*, *nnet* and *caret* available for R > 3.6 in the standard package repository.

Each model was tuned via a grid optimization, when appropriate for the machine learning, and parameters/models were chosen to maximize performance on a ten-fold cross validation using Matthew’s correlation coefficient (MCC). Grids were implemented using the *caret* R packages, where for SVM models the gamma and cost parameters were optimized as well as the classification methods, for NN the network size and the decay parameters were optimized, and for RF the number of trees and number of iterations. Considering the unbalanced dataset, i.e. far more inactive chemicals, under-sampling methods were applied via random selection of inactive chemicals to yield a ratio of 70% inactive and 30% active chemicals. Each model was built ten times with a different inactive set to cover the full set of chemicals. Model performance was reported as a mean with associated standard deviation on the ten repetitions for the training set, the cross-validation, and the external test set.

All InterPred models developed here are available in R environment format on the website: https://sandbox.ntp.niehs.nih.gov/interferences.

#### Performance criteria

The model quality was estimated using five statistical criteria: the accuracy (Acc) or correct classification rate, the specificity (Sp) and sensitivity (Se), i.e. ability of the model to predict active and inactive chemicals respectively, the balanced accuracy (acc_b_, average of sensitivity and specificity) and the Matthew’s correlation coefficient (MCC). MCC is an overall prediction metric (ranging from −1: less than random, to 1: perfect prediction, where 0 corresponds to random) that considers the imbalance ratio between active and inactive chemicals in the data set.

$$\begin{array}{ccc}accuracy & = & \frac{TP+TN}{TP+TN+FP+FN}\\ sensitivity & = & \frac{TP}{TP+FN}\\ specificity & = & \frac{TN}{TN+FP}\\ balanced\,accuracy & = & \frac{sensitivity+specificity}{2}\\ MCC & = & \frac{TP\ast TN-FP\ast FN}{\sqrt{(TP+FP)(TP+FN)(TN+FP)(TN+FN)}}\end{array}$$

True positive (TP) refers to the number of active chemicals that are correctly classified, false negative (FN) is the number of active chemicals that are incorrectly classified, true negative (TN) is the number of inactive chemicals that are correctly classified, and false positive (FP) is the number of inactive chemicals that are incorrectly classified.

Scripts used and developed for this study are available in the GitHub platform https://github.com/ABorrel/interferences.

## Results

To quantify chemical interference in luminescence and autofluorescence assays, five assays with thirteen readouts in total were run. One assay measured firefly luciferase (Fluc) inhibition under cell-free conditions and four assays, consisting of two cell types (HepG2 and HEK-293) and two culture conditions (cell-based and cell-free using culture medium only), measured autofluorescence. Each autofluorescence assay had three fluorescent channel readouts (blue, green and red with filters of emission/excitation equal to 485/535 nm, 405/460 nm, and 540/590 nm, respectively). The Tox21 chemical library of 8305 unique structures were screened in quantitative high-throughput format; see Methods for details.

### Tox21 chemical library

The Tox21 chemical library is composed of 9,667 substances and 8,305 unique structures excluding mixtures and ions. The Tox21 library was built from a federal cross-agency effort covering a large chemical space as discussed in^16,42,43^, and includes diverse chemical groups e.g. pesticides, antimicrobials, water contaminants, industrial chemicals, high production volume chemicals, endocrine disruptors, FDA food additives, fragrances, plasticizers and drugs. The library contains many industrial chemicals prioritized based on potential for human exposure, which are not designed to be bioactive and therefore have a higher potential to interfere with the assays because they have not been filtered out by medicinal chemistry principles used in developing small molecule libraries.

**Figure 1:**
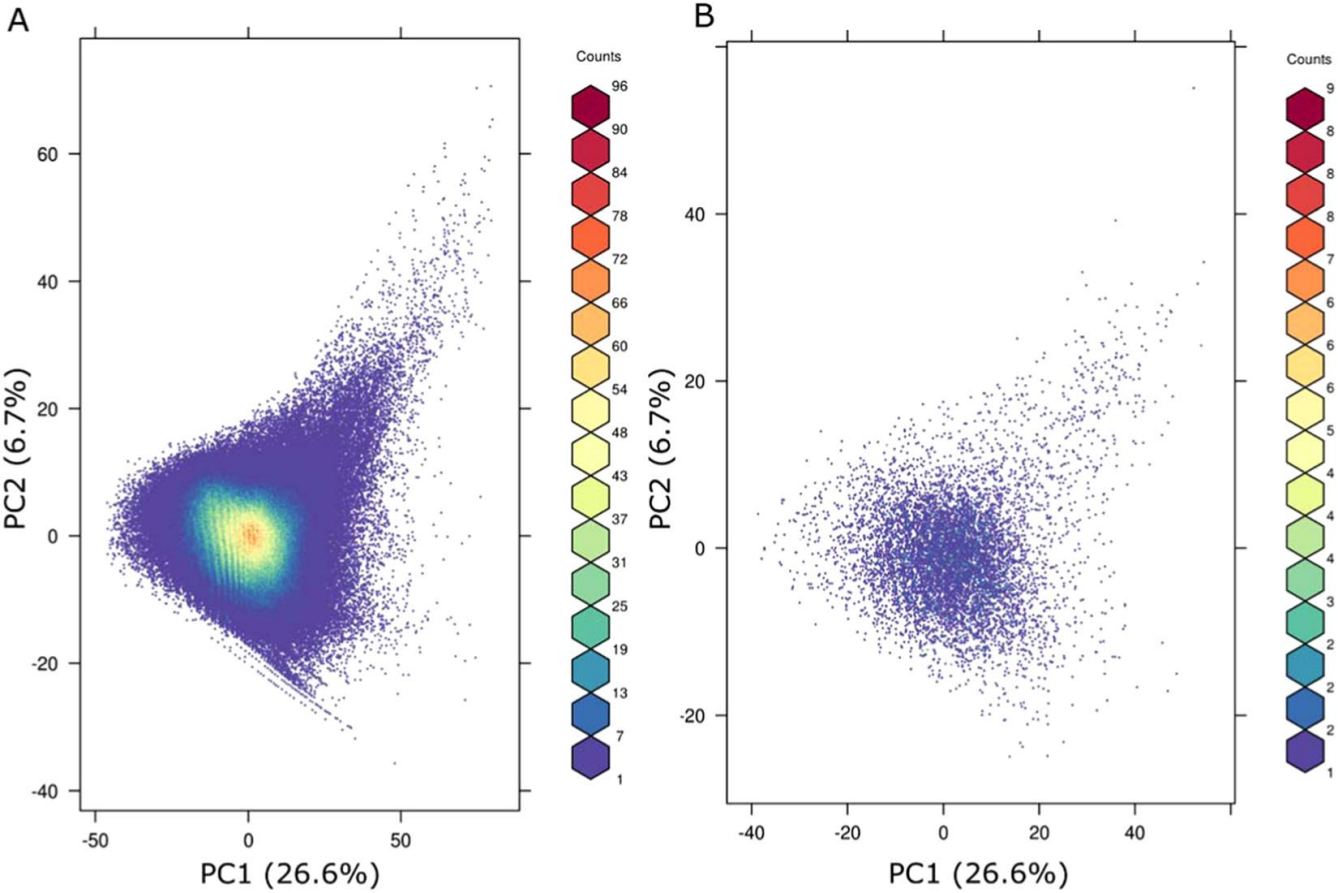
Principal component analysis of 596,526 chemicals (QSAR ready structures) from the DSSTOX database, defined using 1D and 2D descriptors from RDKit library, see methods, covering 33.3% of structural variability. (**A**) represents the projection on the map of the DSSTOX chemicals and (**B**) the 8,305 unique structures from the Tox21 chemical library, showing similarly broad structural coverage.

To examine the chemical coverage of the Tox21 library, chemicals were projected on a Principal component Analysis (PCA) defined using the Distributed Structure-Searchable Toxicity (DSSTox) database covering the largest curated environmental chemical library with more that 800,000 chemicals. Figure 1 shows that the Tox21 chemicals are well distributed on the PCA defined from the DSSTOX using 1D and 2D molecular descriptors, see Methods, and provide broad coverage of the structural landscape

### Reference chemical response curves

**Figure 2:**
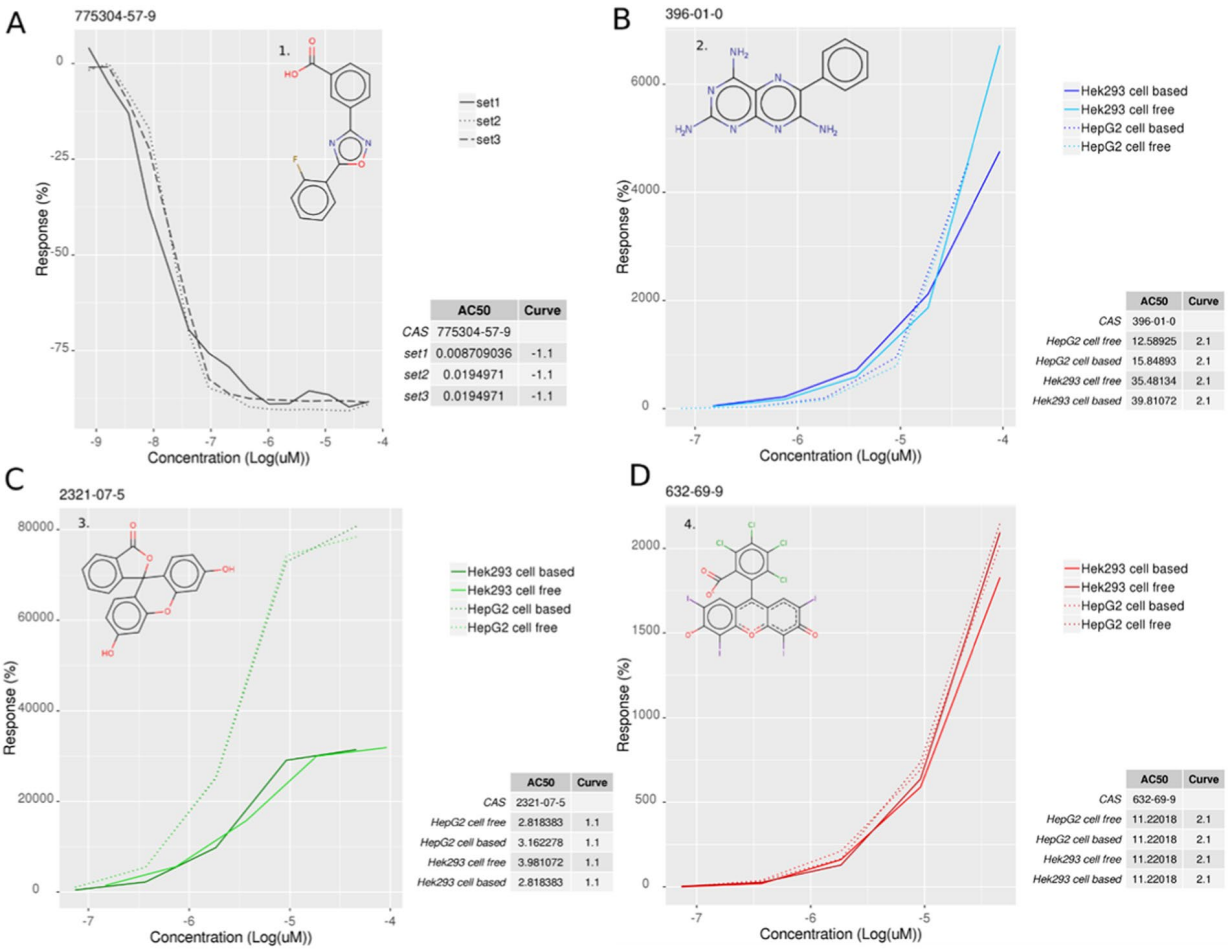
Examples of response curves for positive control chemicals: (**A**) Ataluren - PTC124 (1. 775304-57-4) in luciferase inhibition assay (**B**) Tiramterene (2. 396-01-0) in autofluorescence assay, blue spectrum, (**C**) Fluorescin (3. 2321-07-5) in autofluorescence assay, green spectrum and (**D**) Rose bengal sodium (4. 632-69-9) in autofluorescence assay, red spectrum.

Figure 2A shows the response curve for Ataluren (PTC-124; structure shown in insert panel 1) the reference positive control chemical for the luciferase inhibition assay. PTC-124 has an 3,5-diaryl oxadiazole scaffold ramified with a m-carboxylate which binds Fluc in the ATP active site. The interaction modifies the α-phosphate of ATP through a SN2 displacement reaction and inhibits Fluc^9,44^. The response curve shows rapid signal reduction with an estimated IC_50_ (based on triplicate measurements) below 20 nM. The response curve class was −1.1, which corresponds to a complete inhibition response, see Methods and Supporting Information Table S1 ^18^.

For autofluorescence assays, reference chemicals were the fluorophores Tiramterene (insert panel 2), fluorescein (3) and rose bengal (4) for blue, green and red channels, respectively. Response curves are presented in Fig. 2B–D for each of the four assay conditions (HEK-293 cell-based, HEK-293 cell-free, HepG2 cell-based, and HepG2 cell-free). All reference chemicals response curves showed a significant concentration-dependent fluorescence increase. The signal was stronger for the green channel with AC_50_s <4 µM (curve class 1.1, complete activation response). For the blue and red channels, the AC_50_s were below 40 µM and 12 µM, respectively, with response curve classes equal to 2.1, corresponding to an incomplete response (due to lack of second asymptote) but with >80% efficacy.

### Assay activity summary

The Tox21 chemical library of 8305 different structures was screened using autofluorescence and luciferase inhibition assays to directly measure technology interference. Table 1 summarizes the number of active chemicals by assay. A chemical was defined as active if it passed all of the filters, i.e. AC_50_/IC_50_ cutoff, curve class, and efficacy for luciferase and autofluorescence, and cytotoxicity cutoffs for autofluorescence only. Supporting information Table S2 contains the dataset before and after filtering. For luciferase inhibition assays 6.6% of the chemicals were found to be active, with an average IC_50_ of 28.3 +/− 19.1 µM. For autofluorescence assays, the blue channel had the highest number of active chemicals with between 2.5 to 2.7% of the library depending on cell types and culture conditions. In the green channel between 0.5 to 0.9% of the chemicals were found to be active and between 0.4 to 0.5% in the red channel. The average AC_50_ ranged from 16.5 to 22.3 µM depending on the channel and conditions for autofluorescence and 28.3 for luciferase.

**Table 1:** Assays results summary: percentage of active chemicals computed based on the 8,305 chemicals tested from Tox21.

Assays	Cell culture	Type of AC_50_/IC_50_	# of actives post-filtering	% active	Mean, SD AC_50_/IC_50_ (µM)
Luciferase		Luciferase	552	6.6	28.3 +/− 19.1
Auto-fluorescence	HepG2	Cell based blue	216	2.6	19.8 +/− 14.5
		Cell based green	80	0.9	22.1 +/− 16.2
		Cell based red	39	0.5	16.5 +/− 13.0
		Cell free blue	209	2.5	21.5 +/− 14.3
		Cell free green	80	0.9	19.5 +/− 12.1
		Cell free red	37	0.4	20.5 +/− 14.8
	HEK-293	Cell based blue	210	2.5	22.3 +/− 13.6
		Cell based green	56	0.7	20.0 +/− 13.0
		Cell based red	33	0.4	18.7 +/− 13.9
		Cell free blue	224	2.7	20.7 +/− 13.8
		Cell free green	44	0.5	19.0 +/− 16.1
		Cell free red	34	0.4	19.2 +/− 12.9

**Figure 3:**
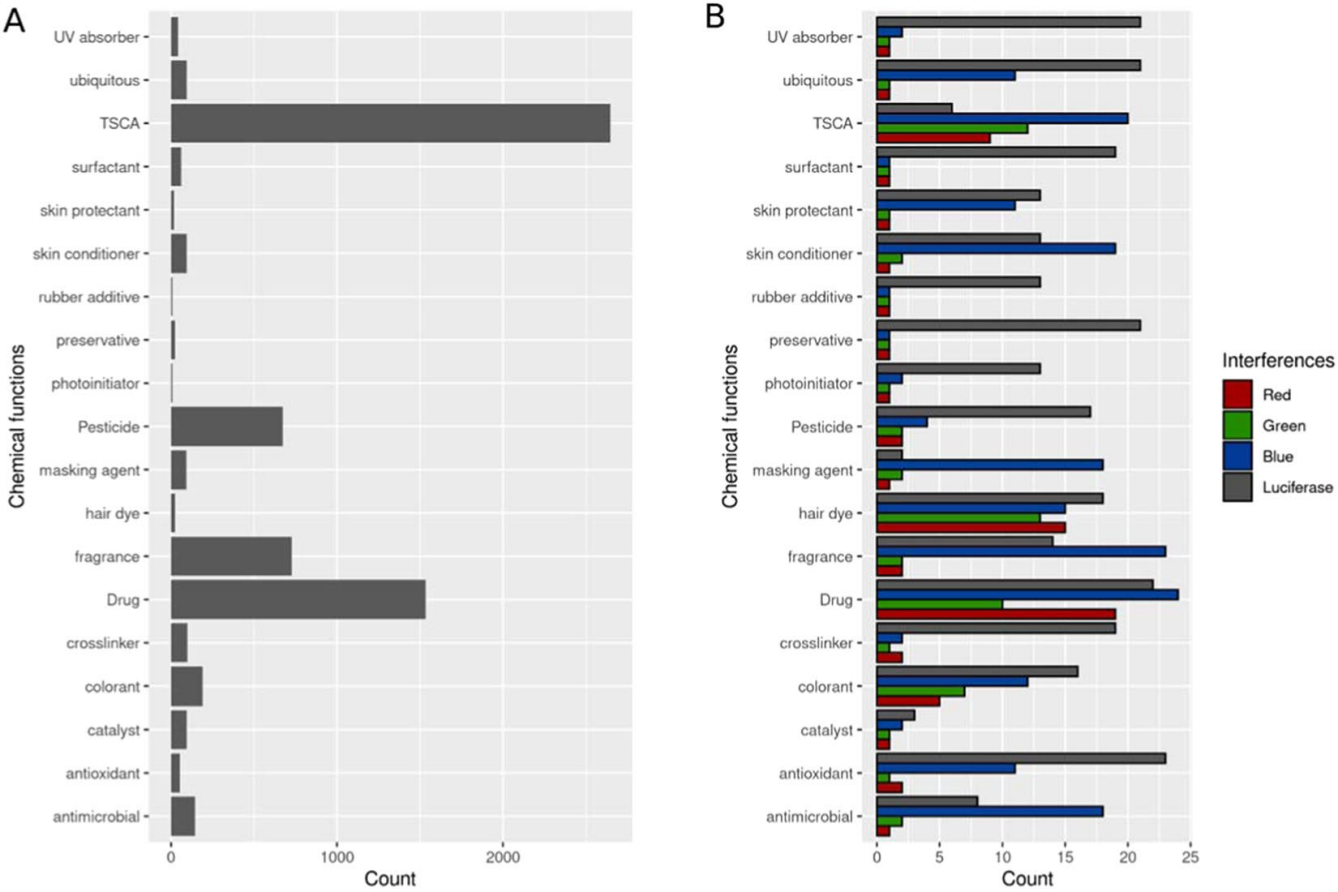
Classification of interference chemicals for the 19 most populated chemical classes (from 80 classes). (**A**) Chemical counts for the 4950 chemicals classified from Tox21 chemicals library. (**B**) Top chemical classes with interference activity for luciferase inhibition and blue, green and red channel autofluorescence.

To characterize chemical interference activity by substance type and use case, the Tox21 chemical library was classified using the consumer products database^45^, the Toxic Substances Control Act chemical list (TSCA), and approved drugs lists available in the EPA chemical dashboard (https://comptox.epa.gov/dashboard). Based on these resources, 4950 of the 8305 unique chemicals included in the Tox21 chemical library could be classified into 80 classes. The most populated classes of active chemicals are represented in Fig. 3. Interferent chemicals are found independently of technology or condition in various chemical classes including chemicals with light absorption properties i.e. UV absorber (enriched for luciferase inhibition), hair dye (enriched for autofluorescence), photo initiator and colorants. Drugs and TSCA classes were also found to be enriched in interference chemicals. Specifically, luciferase interferents were found mostly in preservative, pesticide and antioxidant chemical classes. Chemicals causing autofluorescence in the blue channel were found in skin conditioner, antioxidant and fragrance classes, while red and green channel interferents were enriched for drugs, TSCA and hair dye classes.

**Figure 4:**
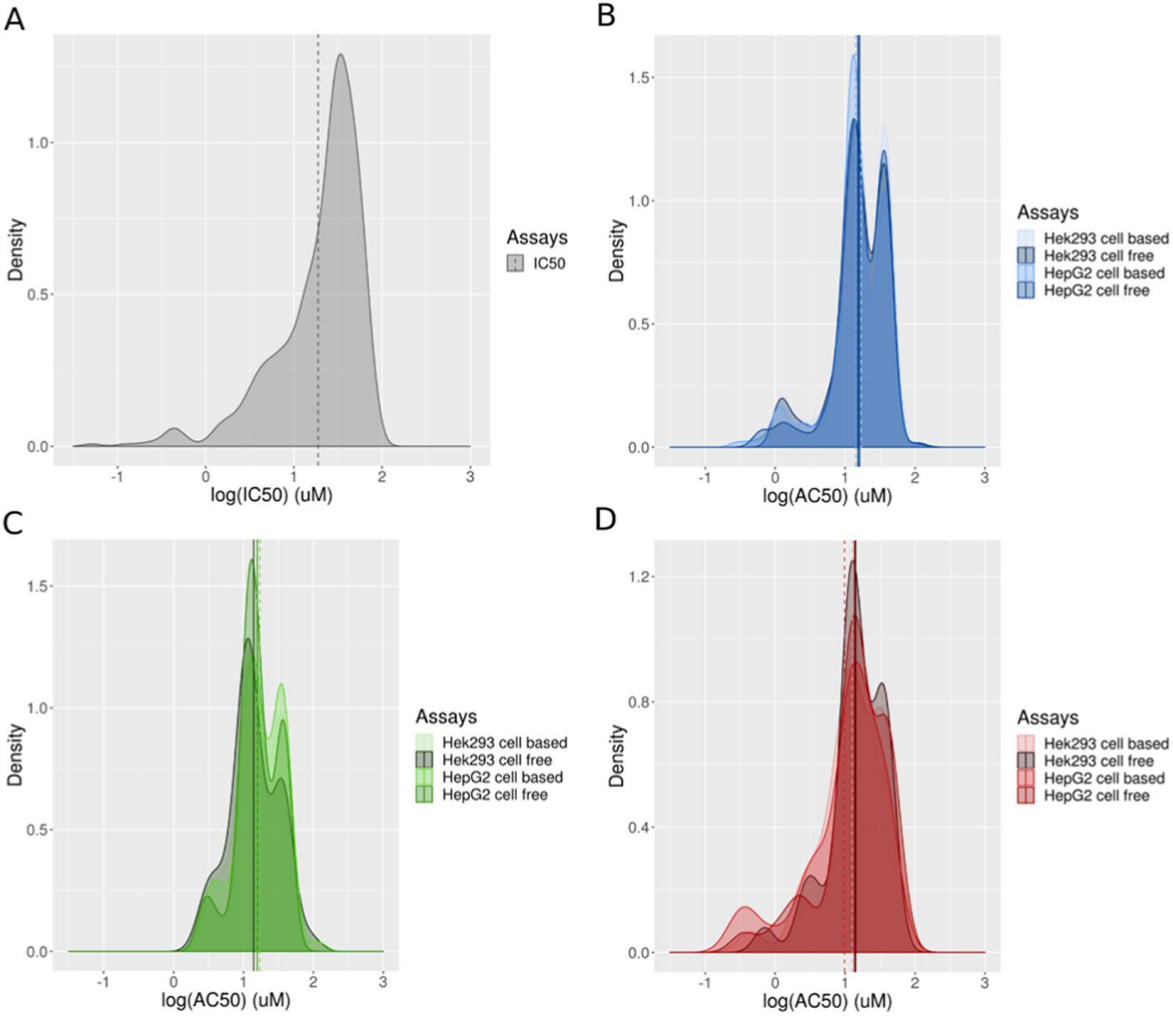
Distributions of (**A**) log(IC_50_) (µM) for luciferase inhibition assay; and (**B**–**D**) log(AC_50_) (µM) for autofluorescence assays using HepG2 and HEK-293 culture cells and cell-free (medium only) conditions. AC_50_ (µM) were measured for the autofluorescence assays in the (**B**) blue, (**C**) green and (**D**) red spectrum. The median of each distribution is represented using a vertical line.

The distribution of activity of selected active chemicals for each assay is shown in Fig. 4 as a density plot. For luciferase inhibition assays, the IC_50_ distribution (Fig. 4A) shows a density peak around 1.5 log(µM) and specific chemical inhibitory concentrations distributed below. The distributions of active chemicals in autofluorescence assays (Fig. 4B–D) appear to be unaffected by any combination of cell type and culture conditions. Interestingly, the activity density plots for all three-color wavelengths demonstrate bimodal potency distributions, with two peaks around 1 and 1.6 log(µM), and the majority of AC_50_ values falling below 2 log(µM). The bimodal potency distribution was found for the most populated curve classes distribution as show in Supporting Information Fig. S1. For compounds with incomplete curves, e.g. curves that did not reach a response plateau, the actual AC_50_ could be higher than the highest test concentration, but to avoid getting an extrapolated range of AC_50_s that are not reliable, our curve fitting algorithm tries to restrict the AC_50_ to below the highest test concentration, thus forming the 2nd peak in the distribution.

### Structural activity patterns: luciferase inhibition

From the Tox21 library, 8,065 unique chemicals were extracted following structure curation, and were clustered using a SOM approach and a set of 165 non-correlated and informative 1D-2D descriptors, see methods. Chemicals were clustered in a SOM set with 225 clusters, allowing good segregation of the chemicals with only two clusters empty and one singleton, and two clusters with less than ten chemicals. On average each cluster is composed of 36 +/− 14 chemicals sharing similar structural properties (Supplementary Information Fig. S2).

**Figure 5:**
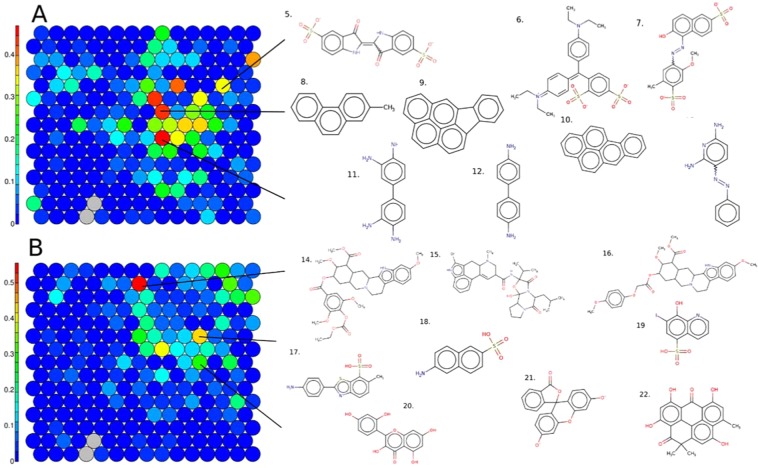
Structure-based SOM on the 8,065 structure-curated Tox21 chemicals, including 225 clusters in total, (**A**) colored using percent active chemicals in luciferase inhibition assays (6.6% of tox21 chemicals), and (**B**) colored using percent active chemicals in autofluorescence assays (4.7% of tox21 chemicals). Left-hand color scales represent the percentage of active chemical by cluster. Example chemical structures for clusters enriched for activity are displayed; cluster 163: 5. 860-22-0, 6. 7, 6. 129-17-9, 7. 25956-17-6; cluster 144: 8. 2531-84-2, 9. 193-39-5, 10. 50-32-8 and cluster 99: 11. 7411-49-6,12. 531-85-1, 13. 136-40-3; cluster 203: 14. 84-36-6, 15. 22260-51-1, 16. 131-01-1; cluster 147: 17. 130-17-6, 18. 93-00-5, 19.547-91-1; cluster 117: 20. 654055-01-3, 21. 518-47-8, 22. 20004-62-0.

The SOM was colored using the percentage of active chemicals found in each cluster, Fig. 5A. Active chemicals were distributed across 154 clusters with 2 +/− 3 active chemicals per cluster, and an average percentage of active chemicals equal to 11 +/− 11% with a maximum equal to 47%. Three clusters that are enriched for chemicals active in the luciferase inhibition assay are highlighted and example structures shown. Cluster 163 had 33% (n = 6) active chemicals, and included three-ring structures ramified with at least one phosphate group and some ketone and alcohol groups, see example structures 5, 6 and 7. Phosphate group substructures can bind the ATP binding site of Fluc and block its activity^9^. Cluster 144 had 43% (n = 4) active chemicals, and included at least five rings structures not ramified, see example structures 8, 9 and 10. Cluster 129, in close proximity on the SOM map, had 44% (n = 10) active chemicals and included at least five connected rings ramified with alcohol, ketone or chlorine for example, structures not shown. Cluster 99, had 47% (5) active chemicals, where structural scaffolds included biphenyl groups (example structures 11, 12) or a diphenyldiazene (13) ramified primary amines groups (11, 12 and 13).

### Structural activity patterns: autofluorescence

The same structure-based SOM run on the entire Tox21 library was then colored according to the percentage of active chemicals by cluster on all of the autofluorescence assays merged together, Fig. 5B. The set of active auto fluorescent chemicals was composed of 379 chemicals distributed across 134 clusters with 3 +/− 2 active chemicals per cluster and an average percentage of active chemicals of 9.2 +/− 8.8%. Three clusters that are enriched for active chemicals in the autofluorescence assays are highlighted and example structures shown.

Cluster 203 had 56% (n = 10) active chemicals and included large chemicals with more than 6 rings (example structures 14, 15 and 16). Chemicals in cluster 147 (44% active chemicals, n = 8) contained two conjugated rings ramified with one phosphate and, for example, primary amine (17, 18) or iodine (19). Finally, cluster 117 had 30% (n = 9) active chemicals and contains structure with not more than three conjugated rings ramified with alcohol groups. (example structures 20, 21, and 22).

**Figure 6:**
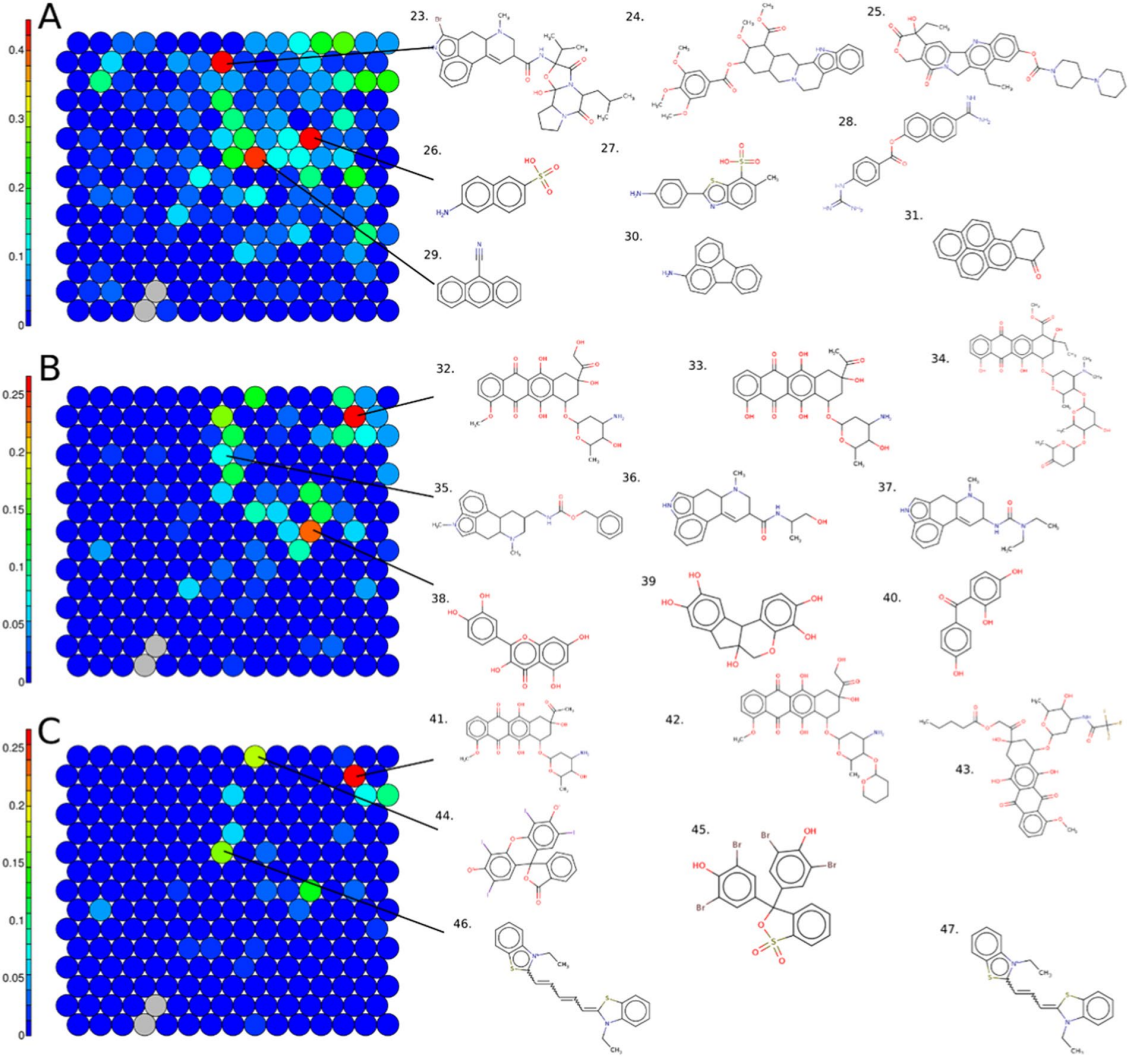
Structure based SOM on the 8,065 Tox21chemicals, colored by the percentage of active chemicals by cluster for autofluorescence assays in color channels: (**A**) blue, (**B**) green and (**C**) red. Example chemical structures for clusters enriched for activity are displayed; cluster 203: 23. 25614-03-3, 24. 131.01-1, 25. 100286-90-6; cluster 147: 26. 93-00-5, 27. 130-17-6, 28. 82956-11-4; cluster 129: 29. 1210-12-4, 30. 2693-46-1, 31. 3331-46-2; cluster 209: 32. 25316-40-9, 33. 50935-04-1, 34. 75443-99-1; cluster 173: 35. 17692-51-2, 36. 60-79-7, 37. 159701-44-7; cluster 117: 38. 6151-25-3, 39. 517-28-2, 40. 1470-79-7; cluster 209: 41. 25316-40-9, 42. 72496-41-4, 43. 56124-62-0; cluster 219: 44. 16423-68-0, 45. 115-39-9; cluster 143: 46. 514-73-8, 47. 905-97-5.

The SOM was then colored by percentage of active chemical by color channel corresponding to blue, green, and red wavelengths, Fig. 6A–C, respectively. The 379 active chemicals in autofluorescence assays were divided into 315 actives for the blue, 95 for the green and 40 for the red channel, see Table 1.

From the three clusters discussed above, none appear to be specific for one color and only cluster 203 was found to also be enriched in the blue channel (56% active chemicals, n = 10). Additional example structures are shown (23, 24 and 25) which have a large scaffold including more than six rings ramified mostly with oxygen groups, e.g. alcohol or ketone.

Structural clusters with specific enrichment for the blue channel include 147 and 129 with 44% and 40% (8 and 10 chemicals) active in each cluster, respectively. Cluster 147 is composed of chemicals with sulfonic acids (example structures 26, 27) or a guanidium substructure (28) connected to an aromatic ring such as naphthalene or benzene. Cluster 129 includes chemicals with a small scaffold constituted by one group of several connected aromatic rings, e.g. anthracene (three aromatic cycles, 29), fluoranthene (30), or pyrene (four cycles, 31).

For the green channel, cluster 209 is the cluster with the highest percentage of active chemicals with 27% actives representing eight chemicals. Active chemicals are composed of at least one unsaturated ring ramified with oxygen groups such as alcohol, ketone or carboxylic acid (example structures 32, 33 and 34), and do not include heteroatoms other than oxygen and carbon. Cluster 173 is also displayed in Fig. 6, with only 8% actives on the green channel but representing five chemicals, all of which include a diazatetracyclohexadeca pentaene and terminal chain composed by nitrogen and oxygen group (example structure 35, 36 and 37). Cluster 117 is also enriched for autofluorescence on the green wavelength with seven actives representing 23% of the full cluster, covering aromatic rings ramified with only alcohol or ketone group (example structures 38, 39, and 40). These chemicals are structural analogues of the fluorescein molecule with a high degree of scaffold similarity.

In the red channel cluster 209 was again enriched with 27% actives, corresponding to eight chemicals. All of the active chemicals in this cluster were autofluorescent in both the green and red channel and included at least one unsaturated ring ramified with oxygen groups such as alcohol, ketone or carboxylic acid (additional example structures 41, 42 and 43).

Cluster 194 included two chemicals representing 18% (n = 4) of the cluster actives with structure composed by three aromatics ramified with iodine (44), bromine (45) and also chlorine, not shown. Finally, cluster 143 also included three active chemicals representing 17% of the cluster. Structurally, chemicals include on both extremities a benzothiazole connected with an unsaturated chain (example structures 46 and 47), and no oxygens.

### Autofluorescence patterns among channels and culture conditions

**Figure 7:**
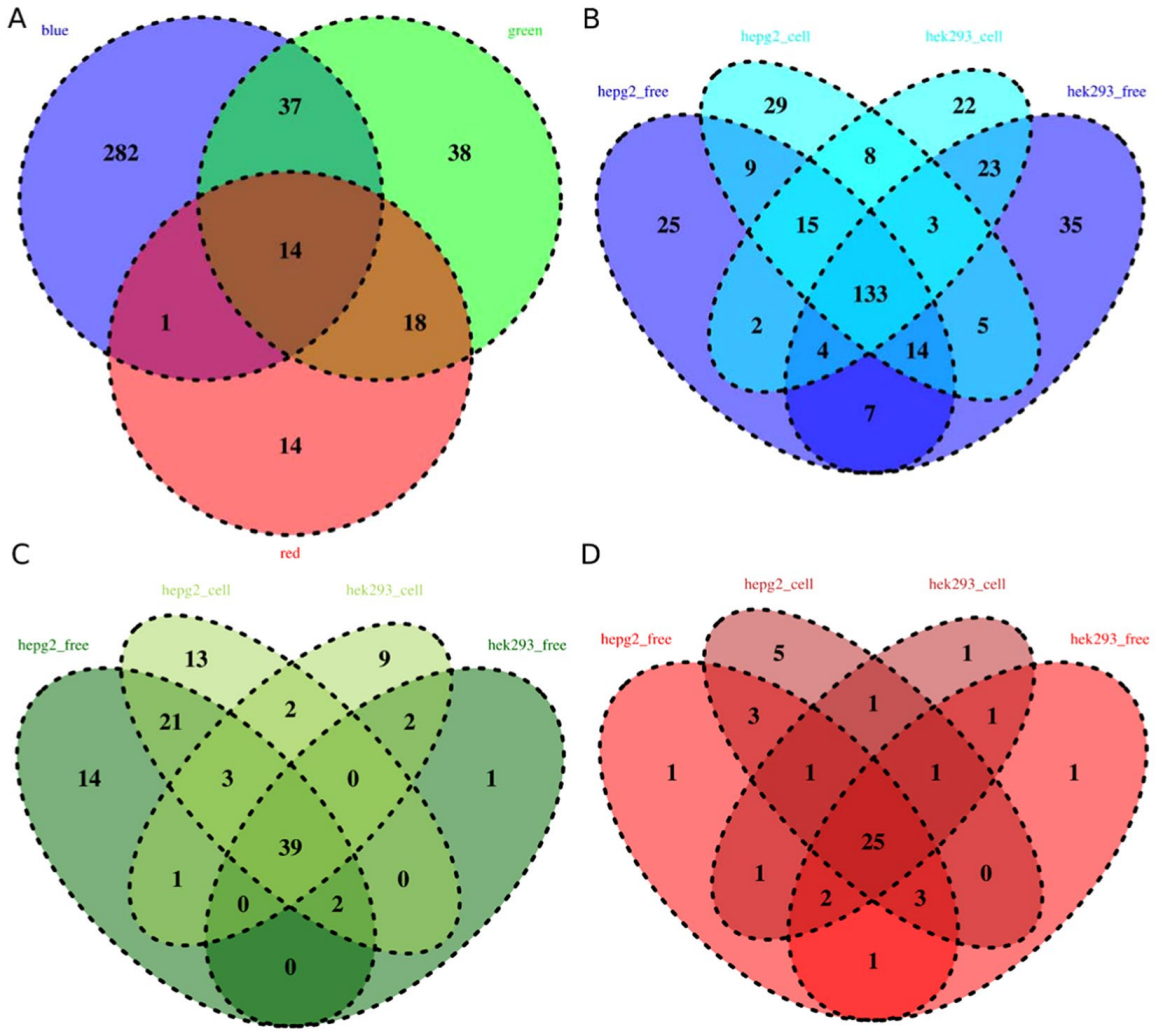
Venn diagrams of active chemicals for autofluorescence assays, (**A**) comparing different color channels, (**B**) considering only the blue channel and comparing cell lines/culture conditions, (**C**) considering only the green channel and comparing cell lines/culture conditions, and (**D**) considering only the red channel and comparing cell lines/culture conditions. For specific color channels cell culture conditions are represented as cell based (hepg2_cell and hek293_cell) and cell free, culture medium only (hepg2_free and hek293_free).

For each color channel (blue, green, and red), autofluorescence activity was measured using two cell types (HepG2 and HEK-293) and two culture conditions, cell-free (culture medium only) and cell-based. As shown with the SOM, some chemicals demonstrated activity across color channels. Figure 7 quantifies the overlap between autofluorescent chemicals by color channel (Fig. 7A) and within each wavelength by cell line/culture condition (Fig. 7B–D).

Only 3.5% of the 404 autofluorescent chemicals were active across all cell culture conditions and color wavelengths, Fig. 7A. The 14 chemicals are shown in Supporting Information Table S3. Structurally these chemicals included usually a large scaffold with a complex aromatic ring arrangement composed of more than 3 rings. Figure 7B–D defines the overlap of active chemicals among cell culture conditions, within a color channel. A chemical that is autofluorescent in every combination of cell culture condition can be considered strongly interferent with the color channel with a high confidence; however, these cases represented only a portion of the active chemicals: 40% of actives in the blue, 36% in the green channel and 53% for the red channel. The overlap among autofluorescence assays and the luciferase inhibition assay is shown in Supplemental Fig. S3.

### Hierarchical clustering on active chemicals

**Figure 8:**
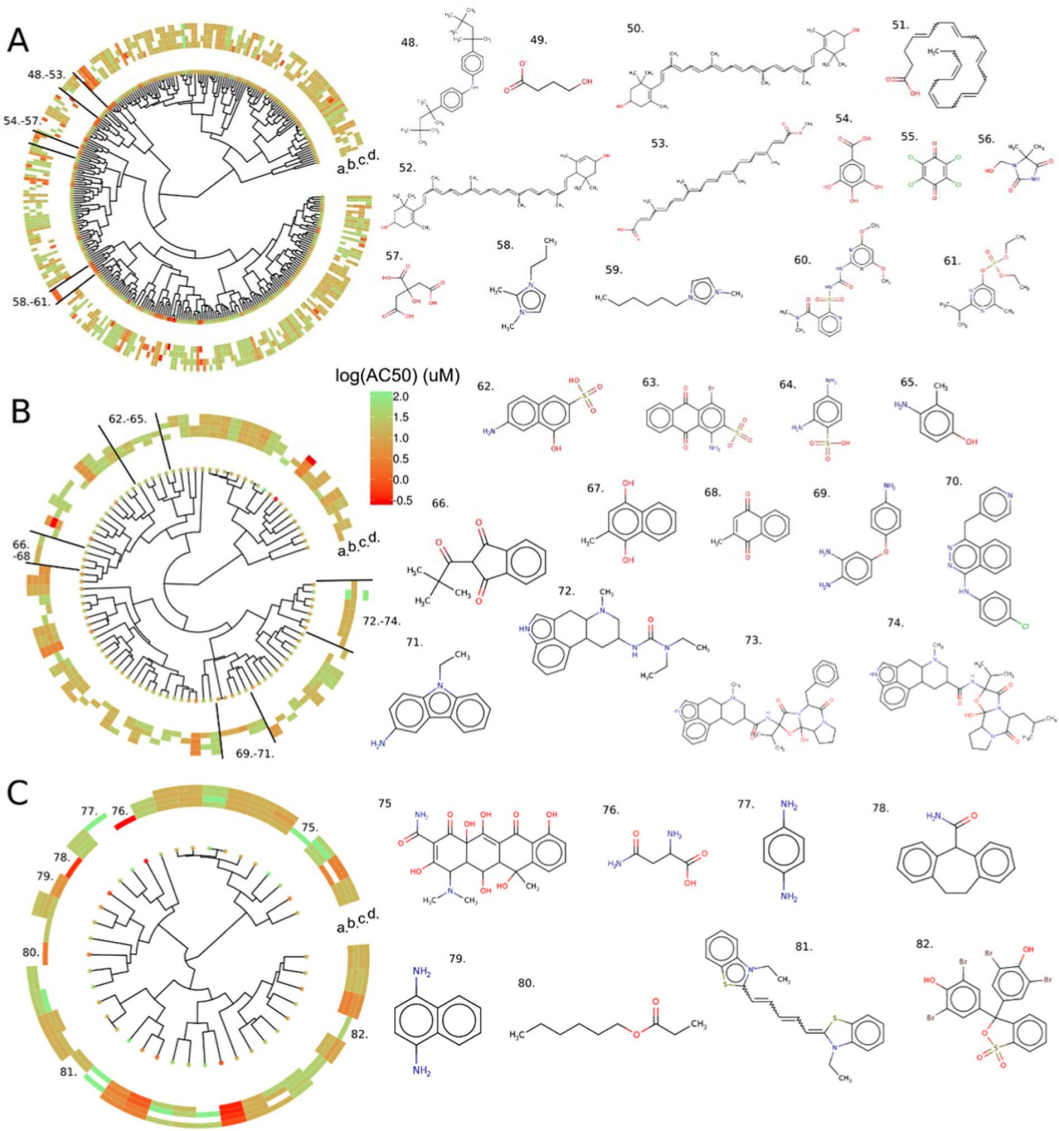
Hierarchical clustering of active chemicals in autofluorescence assays for (**A**) blue, (**B**) green and (**C**) red channel. Hierarchical clustering is realized using a Euclidean distance computed from a set of non-redundant molecular descriptors and using Ward segregation, see methods. Potency in terms of AC_50_ for each chemical is represented using a color scale from red to green and for the four cell culture conditions, a. Hepg2 cell based, b. Hepg2 cell free, c. HEK-293 cell based and d. HEK-293 cell free. Some chemical structures in active chemical clusters specific to one cell culture/condition are represented; 48. 15721-78-5, 49. 508-85-2, 50. 144-68-3, 51. 6217-54-5, 52. 127-40-2, 53. 6983-79-5, 54. 149-91-7, 55. 118-75-2, 56. 118-75-2, 57. 77-92-9, 58. 218151-78-1, 59. 85100-78-3, 60. 111991-09-4, 61. 962-58-3, 62. 9-51-7, 63. 6258-06-6, 64. 88-63-1, 65. 2835-99-6, 66. 83-26-1, 67. 481-85-6, 68. 58-27-5, 69. 6264-66-0, 70. 212141-51-0, 71. 132-32-1, 72. 159701-44-7, 73. 17479-19-5, 74. 25447-66-9, 75. 6153-64-6, 76. 5794-24-1, 77. 106-50-30, 78. 7199-29-3, 79. 2243-61-0, 80. 2445-76-3, 81. 54646-38-7, 82. 115-39-9.

The influence of four different cell culture conditions (HepG2 and HEK-293 cell lines with cell-based and cell-free conditions) on chemical activity in the autofluorescence assays was investigated across the three-color channels. To identify clusters of chemicals specifically active for one cell culture or condition for different color channels, hierarchical clustering was performed where active chemicals sharing similar physico-chemical or topological properties were clustered together. The clustering is presented using a circular dendrogram, for each color channel and cell culture condition (Fig. 8A–C) and colored by potency. Chemicals specifically active for certain experimental conditions are highlighted and structures shown. Hierarchical clustering of autofluorescent chemicals across all twelve combinations of cell culture conditions and color channels is shown via circular dendrogram in Supporting Information Fig. S4, and for all active chemicals in the luciferase inhibition assay in Fig. S5.

Among chemicals active within wavelengths, certain chemical features were found to be associated with activity for one cell culture condition. Chemicals 62, 63, 64, 65 and 79 were found active specifically on the HepG2 culture. These chemicals have small structural scaffolds, less than 20 atoms with not more than three conjugated rings such as benzoindole or naphthalene. Similarly, chemicals 49, 57, 66, 67, 68, 69 and 77 are active only on HEK-293 and share one benzene ramified with primary amine or alcohol groups. These structural scaffolds may drive interference with the culture conditions rather than the color channels.

Specific to the blue channel, chemicals 50, 51, 52 and 53, containing unsaturated chains ramified with several methyl groups, interact specifically with the HEK-293 cell-based and cell-free condition. Chemicals 58 and 59 composed of an imidazole ring connected with an aliphatic chain, were active only in the HEK-293 cell-free condition and chemicals 60 and 61 composed of a pyrimidine and a phosphate group are also found specifically active on HEK-293 cell-free condition.

Finally, some of the chemicals represented in Fig. 8 (structures 48, 54, 55, 56, 72, 73, 74, 75, 79, 80 and 81) included chemical substructures important for fluorescence, e.g. high number of aromatic rings and are close structural analogues of chemicals already discussed in the SOM results.

### QSAR interference classification models

As shown using unsupervised statistical approaches, chemicals that actively inhibit luciferase share similar structural chemical properties and, separately, those that interfere with autofluorescence assays have common features, in some cases specific to interference patterns driven by assay platform, color channel, cell or condition. Based on structural and physico-chemical properties, quantitative structure–activity relationships (QSAR) models were developed to predict the likelihood of chemical-assay interference. Multiple machine learning approaches (see Methods) were used to predict luciferase inhibition, autofluorescence in any color channel across cell culture conditions, autofluorescence individually by color channel regardless of conditions, and autofluorescence by color channel and cell culture conditions uniquely. All model building steps, including undersampling from the over-represented set of inactives in the Tox21 dataset, training, cross-validation, and testing, were repeated ten times to ensure that all chemicals were incorporated in the process, and the mean and standard deviation of each performance criterion across all ten iterations were reported for each machine learning approach.

First, QSAR models were built to predict luciferase inhibition. After data processing, each full set of chemicals for model building was composed of 1,724 chemicals (30% actives, 70% inactives; Table 2). The RF model achieved the best performance, with a cross-validation MCC equal to 0.557 +/− 0.016 and test set MCC of 0.571 +/− 0.045, confirming the stability of the model and the limited overfitting in spite of the high performance on the training set (MCC of 0.996 +/− 0.002). MCC is an overall prediction metric (where 0 is random and 1 is perfect) characterizing correlation between predicted and measured values that considers the imbalance ratio between active and inactive chemicals in the data set (see Methods).

**Table 2:** Performance of QSAR classification models for luciferase inhibition.

	Luciferase assay				
***10-fold cross-validation (full set, n*** = ***1724)***					
***Method***	***Acc***	***Acc***_***b***_	***Sp***	***Se***	***MCC***
*RF*	0.828 +/− 0.006	0.752 +/− 0.0095	0.931 +/− 0.002	0.573 +/− 0.017	0.557 +/− 0.016
*SVM-linear*	0.804 +/− 0.05	0.715 +/− 0.011	0.923 +/−0.007	0.507 +/− 0.015	0.486 +/− 0.014
*SVM-radial*	0.719 +/− 0.002	0.512 +/− 0.0025	0.997 +/− 0.001	0.027 +/− 0.004	0.109 +/− 0.014
*SVM-sigmoid*	0.669 +/− 0.011	0.563 +/− 0.012	0.813 +/− 0.011	0.312 +/− 0.014	0.136 +/− 0.025
*LDA*	0.806 +/− 0.005	0.744 +/− 0.0125	0.89 +/− 0.007	0.598 +/− 0.018	0.51 +/− 0.015
*CART*	0.783 +/− 0.01	0.7055 +/− 0.014	0.888 +/− 0.011	0.523 +/− 0.017	0.442 +/− 0.026
*NN*	0.783 +/− 0.011	0.7185 +/− 0.024	0.869 +/− 0.011	0.568 +/− 0.037	0.453 +/− 0.031
***Fitting (training set, n*** = ***1464)***					
***Method***	***M Acc***	***Acc***_***b***_	***Sp***	***Se***	***MCC***
*RF*	0.998 +/− 0.001	0.997 +/− 0.002	0.999 +/− 0.001	0.995 +/− 0.003	0.996 +/− 0.002
*SVM-linear*	0.822 +/− 0.003	0.738 +/− 0.013	0.935 +/− 0.006	0.542 +/− 0.02	0.538 +/− 0.01
*SVM-radial*	0.973 +/− 0.004	0.953 +/− 0.006	1 +/− 0	0.906 +/− 0.012	0.934 +/− 0.008
*SVM-sigmoid*	0.638 +/− 0.01	0.531 +/− 0.019	0.783 +/− 0.012	0.279 +/− 0.026	0.066 +/− 0.026
*LDA*	0.848 +/− 0.007	0.7935 +/− 0.011	0.923 +/− 0.006	0.664 +/− 0.016	0.617 +/− 0.018
*CART*	0.137 +/− 0.012	0.1925 +/− 0.0275	0.062 +/− 0.01	0.323 +/− 0.045	−0.653 +/− 0.032
*NN*	0.837 +/− 0.022	0.798 +/− 0.036	0.892 +/− 0.026	0.704 +/− 0.046	0.602 +/− 0.051
***External validation (test set, n*** = ***258)***					
***Method***	***Acc***	***Acc***_***b***_	***Sp***	***Se***	***MCC***
*RF*	0.835 +/− 0.024	0.7645 +/− 0.041	0.925 +/− 0.026	0.604 +/− 0.056	0.571 +/− 0.045
*SVM-linear*	0.811 +/− 0.032	0.727 +/− 0.0385	0.918 +/− 0.029	0.536 +/− 0.048	0.502 +/− 0.064
*SVM-radial*	0.724 +/− 0.037	0.51 +/− 0.01	0.994 +/− 0.004	0.026 +/− 0.016	0.079 +/− 0.067
*SVM-sigmoid*	0.667 +/− 0.021	0.56 +/− 0.05	0.807 +/− 0.031	0.313 +/− 0.069	0.128 +/− 0.068
*LDA*	0.802 +/− 0.027	0.7405 +/− 0.036	0.879 +/− 0.027	0.602 +/− 0.045	0.495 +/− 0.059
*CART*	0.207 +/− 0.023	0.275 +/− 0.033	0.12 +/− 0.013	0.43 +/− 0.053	−0.467 +/− 0.045
*NN*	0.786 +/− 0.031	0.735 +/− 0.048	0.845 +/− 0.033	0.625 +/− 0.063	0.468 +/− 0.072

Each model building process was repeated 10 times with distinct data segregation and inactive under sampling from the entire Tox21 dataset, and the mean (M) and the standard deviation (SD) of each performance criterion are reported, Acc: accuracy, Acc_b_: balanced accuracy, Sp: specificity, Se: sensitivity and MCC: Matthew Coefficient Correlation, see methods.

LDA, NN and SVM with a linear kernel (SVM-linear) exhibited close performance to the RF QSAR models, with cross-validation MCC equal to 0.51 +/− 0.015, 0.453 +/− 0.031 and 0.486 +/− 0.0014, respectively, but the CART and SVM nonlinear models (SVM-radial and SVM-sigmoid) were weaker (cross-validation MCC of 0.442 +/− 0.026, 0.109 +/− 0.014 and 0.136 +/− 0.025, respectively). In the test set SVM-sigmoid performed better than CART model with MCC equal to 0.128 +/− 0.068 and −0.467 +/− 0.045, respectively.

For autofluorescence assays, QSAR models were first developed to predict activity without distinguishing by color channel or cell culture conditions; performance metrics are reported in Table 3. Only chemicals that were active across all conditions for each color (independently) were used for the active training set. For each modeling iteration, the full set included 507 chemicals (30% actives, 70% inactives). Similar to QSAR models developed for luciferase inhibition, RF gave the best performance with a cross validation MCC equal to 0.564 +/− 0.026 and test set MCC of 0.568 +/− 0.094, and CART, SVMs, NN and LDA QSAR models had lower performance.

**Table 3:** Performance of QSAR classification models for autofluorescence activity.

	Autofluorescence assays				
***10-fold cross-validation (full set, n*** = ***507)***					
***Method***	***Acc***	***Acc***_***b***_	***Sp***	***Se***	***MCC***
*RF*	0.823 +/− 0.01	0.764 +/− 0.020	0.915 +/− 0.012	0.613 +/− 0.027	0.564 +/− 0.026
*SVM-linear*	0.817 +/− 0.011	0.748 +/− 0.0165	0.925 +/− 0.011	0.571 +/− 0.022	0.545 +/− 0.028
*SVM-radial*	0.717 +/− 0.004	0.5365 +/− 0.005	0.999 +/− 0.001	0.074 +/− 0.009	0.224 +/− 0.017
*SVM-sigmoid*	0.697 +/− 0.011	0.6035 +/− 0.0205	0.843 +/− 0.016	0.364 +/− 0.025	0.23 +/− 0.029
*LDA*	0.776 +/− 0.012	0.7385 +/− 0.020	0.834 +/− 0.013	0.643 +/− 0.026	0.475 +/− 0.027
*CART*	0.78 +/− 0.018	0.7265 +/− 0.029	0.863 +/− 0.018	0.59 +/− 0.039	0.468 +/− 0.044
*NN*	0.776 +/− 0.02	0.7315 +/− 0.0375	0.846 +/− 0.029	0.617 +/− 0.046	0.468 +/− 0.045
***Fitting (training set, n*** = ***431)***					
***Method***	***Acc***	***Acc***_***b***_	***Sp***	***Se***	***MCC***
*RF*	0.998 +/− 0.004	0.997 +/− 0.006	0.999 +/− 0.002	0.995 +/− 0.009	0.995 +/− 0.01
*SVM-linear*	0.851 +/− 0.009	0.7895 +/− 0.015	0.945 +/− 0.004	0.634 +/− 0.026	0.633 +/− 0.023
*SVM-radial*	0.975 +/− 0.004	0.9595 +/− 0.006	1 +/− 0	0.919 +/− 0.012	0.942 +/− 0.008
*SVM-sigmoid*	0.667 +/− 0.016	0.57 +/− 0.028	0.816 +/− 0.028	0.324 +/− 0.028	0.154 +/− 0.026
*LDA*	0.921 +/− 0.012	0.902 +/− 0.019	0.952 +/− 0.012	0.852 +/− 0.026	0.812 +/− 0.028
*CART*	0.103 +/− 0.006	0.1305 +/− 0.036	0.062 +/− 0.018	0.199 +/− 0.053	−0.753 +/− 0.018
*NN*	0.884 +/− 0.042	0.8645 +/− 0.052	0.913 +/− 0.049	0.816 +/− 0.055	0.729 +/− 0.091
***External validation (test set, n*** = ***76)***					
***Method***	***Acc***	***Acc***_***b***_	***Sp***	***Se***	***MCC***
*RF*	0.818 +/− 0.041	0.767 +/− 0.079	0.905 +/− 0.048	0.629 +/− 0.109	0.568 +/− 0.094
*SVM-linear*	0.807 +/− 0.043	0.7505 +/− 0.0715	0.906 +/− 0.039	0.595 +/− 0.104	0.537 +/− 0.113
*SVM-radial*	0.705 +/− 0.019	0.537 +/− 0.026	1 +/− 0	0.074 +/− 0.052	0.196 +/− 0.115
*SVM-sigmoid*	0.694 +/− 0.031	0.61 +/− 0.043	0.843 +/− 0.038	0.377 +/− 0.048	0.247 +/− 0.055
*LDA*	0.797 +/− 0.034	0.7735 +/− 0.065	0.834 +/− 0.045	0.713 +/− 0.084	0.54 +/− 0.08
*CART*	0.211 +/− 0.055	0.248 +/− 0.079	0.148 +/− 0.044	0.348 +/− 0.113	−0.508 +/− 0.125
*NN*	0.765 +/− 0.043	0.735 +/− 0.0825	0.816 +/− 0.032	0.654 +/− 0.133	0.463 +/− 0.123

Only chemicals active in any color channel under all cell culture conditions were considered as actives. Each model building process was repeated 10 times with distinct data segregation and inactive undersampling from the entire Tox21 dataset, and the mean and the standard deviation of each performance criterion are reported, Acc: accuracy, Acc_b_: balanced accuracy, Sp: specificity, Se: sensitivity and MCC: Matthew Coefficient Correlation see methods.

Specific QSAR models were then developed for each color channel. A chemical was considered active if it was active on at least one cell culture condition in a particular color channel. Performance metrics are presented in Table 4. The modeling dataset for the blue channel consisted of 1,045 chemicals, for the green channel 339 chemicals, and for the red channel 148 chemicals. As with the previous QSAR models, RF performed better in each case followed by SVM-linear, NN and SVM nonlinear/CART. The highest performing color-specific model was on the red channel, with MCCs equal to 0.691 +/− 0.043 and 0.672 +/− 0.092 on the cross validation and test set, respectively, followed by the green channel with cross validation and test set MCCs of 0.642 +/− 0.025 and 0.651 +/− 0. 085 respectively. Performances are lower on the blue channel with MCCs of 0.431 +/− 0.028 and 0.438 +/− 0.075 for the cross validation and test set. However, in the red channel, the standard deviation associated with the MCC mean is higher than other color channels.

**Table 4:** Performance of QSAR classification models autofluorescence activity specific to wavelength.

	Autofluorescence assays				
**Blue**					
***10-fold cross-validation (full set, n*** = ***1045)***					
*Method*	*Acc*	*Acc*_*b*_	*Sp*	*Se*	*MCC*
*RF*	0.773 +/− 0.011	0.685 +/− 0.02	0.917 +/− 0.013	0.453 +/− 0.027	0.431 +/− 0.028
*SVM-linear*	0.759 +/− 0.009	0.67 +/− 0.011	0.905 +/− 0.01	0.435 +/− 0.012	0.394 +/− 0.022
*SVM-radial*	0.705 +/− 0.003	0.527 +/− 0.0045	0.998 +/− 0.002	0.056 +/− 0.007	0.184 +/− 0.019
*SVM-sigmoid*	0.633 +/− 0.015	0.5475 +/− 0.016	0.772 +/− 0.015	0.323 +/− 0.017	0.101 +/− 0.032
*LDA*	0.737 +/− 0.01	0.6685 +/− 0.014	0.849 +/− 0.008	0.488 +/− 0.02	0.357 +/− 0.026
*CART*	0.728 +/− 0.014	0.656 +/− 0.025	0.845 +/− 0.013	0.467 +/− 0.036	0.333 +/− 0.036
*NN*	0.722 +/− 0.011	0.670 +/− 0.037	0.808 +/− 0.027	0.531 +/− 0.048	0.344 +/− 0.026
***Fitting (training set, n*** = ***888)***					
***Method***	***Acc***	***Acc***_***b***_	***Sp***	***Se***	***MCC***
*RF*	0.998 +/− 0.002	0.9965 +/− 0.004	0.999 +/− 0.001	0.994 +/− 0.006	0.995 +/− 0.005
*SVM-linear*	0.801 +/− 0.005	0.7175 +/− 0.014	0.936 +/− 0.01	0.499 +/− 0.018	0.505 +/− 0.011
*SVM-radial*	0.974 +/− 0.004	0.958 +/− 0.0065	1 +/− 0.001	0.916 +/− 0.012	0.939 +/− 0.01
*SVM-sigmoid*	0.605 +/− 0.021	0.518 +/− 0.024	0.747 +/− 0.027	0.289 +/− 0.021	0.039 +/− 0.04
*LDA*	0.823 +/− 0.008	0.764 +/− 0.014	0.918 +/− 0.006	0.61 +/− 0.022	0.568 +/− 0.019
*CART*	0.144 +/− 0.009	0.188 +/− 0.029	0.072 +/− 0.013	0.304 +/− 0.044	−0.654 +/− 0.024
*NN*	0.799 +/− 0.027	0.7515 +/− 0.07	0.877 +/− 0.048	0.626 +/− 0.092	0.522 +/− 0.065
***External validation (test set, n*** = ***157)***					
***Method***	***Acc***	***Acc***_***b***_	***Sp***	***Se***	***MCC***
*RF*	0.773 +/− 0.025	0.6905 +/− 0.044	0.916 +/− 0.023	0.465 +/− 0.065	0.438 +/− 0.075
*SVM-linear*	0.762 +/− 0.033	0.678 +/− 0.0525	0.91 +/− 0.027	0.446 +/− 0.078	0.411 +/− 0.089
*SVM-radial*	0.698 +/− 0.032	0.5245 +/− 0.014	0.999 +/− 0.003	0.05 +/− 0.025	0.176 +/− 0.045
*SVM-sigmoid*	0.655 +/− 0.025	0.5785 +/− 0.054	0.793 +/− 0.039	0.364 +/− 0.069	0.167 +/− 0.054
*LDA*	0.733 +/− 0.028	0.663 +/− 0.038	0.857 +/− 0.026	0.469 +/− 0.049	0.352 +/− 0.061
*CART*	0.278 +/− 0.021	0.3425 +/− 0.054	0.165 +/− 0.045	0.52 +/− 0.063	−0.334 +/− 0.065
*NN*	0.729 +/− 0.044	0.6765 +/− 0.104	0.826 +/− 0.059	0.527 +/− 0.149	0.363 +/− 0.115
**Green**					
***10-fold cross-validation (full set, n*** = ***339)***					
***Method***	***Acc***	***Acc***_***b***_	***Sp***	***Se***	***MCC***
*RF*	0.856 +/− 0.009	0.7975 +/− 0.022	0.943 +/− 0.009	0.652 +/− 0.034	0.642 +/− 0.025
*SVM-linear*	0.81 +/− 0.011	0.72 +/− 0.0215	0.942 +/− 0.017	0.498 +/−0.026	0.516 +/−0.027
*SVM-radial*	0.719 +/− 0.006	0.5285 +/− 0.005	0.998 +/− 0.002	0.059 +− 0.008	0.193 +/− 0.024
*SVM-sigmoid*	0.689 +/− 0.031	0.5925 +/− 0.0415	0.829 +/− 0.035	0.356 +/− 0.048	0.204 +/− 0.068
*LDA*	0.783 +/− 0.023	0.7605 +/− 0.030	0.817 +/− 0.02	0.704 +/− 0.039	0.503 +/− 0.051
*CART*	0.79 +/− 0.026	0.741 +/− 0.044	0.862 +/− 0.015	0.62 +/− 0.072	0.49 +/− 0.07
*NN*	0.8 +/− 0.024	0.76 +/− 0.0405	0.858 +/− 0.028	0.662 +/− 0.053	0.521 +/− 0.055
***Fitting (training set, n*** = ***288)***					
***Method***	***Acc***	***Acc***_***b***_	***Sp***	***Se***	***MCC***
*RF*	0.998 +/− 0.002	0.997 +/− 0.004	0.999 +/− 0.002	0.995 +/− 0.006	0.995 +/− 0.006
*SVM-linear*	0.871 +/− 0.039	0.8065 +/− 0.064	0.965 +/− 0.012	0.648 +/− 0.116	0.678 +/− 0.099
*SVM-radial*	0.977 +/− 0.008	0.9615 +/− 0.014	0.999 +/− 0.002	0.924 +/− 0.026	0.946 +/− 0.019
*SVM-sigmoid*	0.624 +/− 0.033	0.5305 +/− 0.046	0.76 +/− 0.037	0.301 +/− 0.055	0.063 +/− 0.07
*LDA*	0.976 +/− 0.01	0.9695 +/− 0.014	0.986 +/− 0.012	0.953 +/− 0.016	0.943 +/− 0.024
*CART*	0.096 +/− 0.007	0.1305 +/− 0.041	0.046 +/− 0.023	0.215 +/− 0.059	−0.767 +/− 0.017
*NN*	0.914 +/− 0.026	0.8965 +/− 0.038	0.941 +/− 0.035	0.852 +/− 0.041	0.797 +/− 0.056
***External validation (test set, n*** = ***51)***					
***Method***	***Acc***	***Acc***_***b***_	***Sp***	***Se***	***MCC***
*RF*	0.857 +/− 0.032	0.8075 +/− 0.069	0.932 +/− 0.039	0.683 +/− 0.099	0.651 +/− 0.085
*SVM-linear*	0.823 +/− 0.04	0.7405 +/− 0.0745	0.952 +/− 0.037	0.529 +/− 0.112	0.561 +/− 0.107
*SVM-radial*	0.713 +/− 0.038	0.5285 +/− 0.0225	1 +/− 0	0.057 +/− 0.045	0.168 +/− 0.116
*SVM-sigmoid*	0.674 +/− 0.032	0.5965 +/− 0.088	0.794 +/− 0.055	0.399 +/− 0.121	0.199 +/− 0.087
*LDA*	0.749 +/− 0.048	0.729 +/− 0.094	0.779 +/− 0.064	0.679 +/− 0.124	0.44 +/− 0.11
*CART*	0.211 +/− 0.067	0.2525 +/− 0.114	0.149 +/− 0.078	0.356 +/− 0.15	−0.502 +/− 0.16
*NN*	0.784 +/− 0.068	0.74 +/− 0.1085	0.853 +/− 0.061	0.627 +/− 0.156	0.487 +/− 0.16
**Red**					
***10-fold cross-validation (training set, n*** = ***148)***					
***Method***	***Acc***	***Acc***_***b***_	***Sp***	***Se***	***MCC***
*RF*	0.877 +/− 0.016	0.82 +/− 0.042	0.955 +/− 0.022	0.685 +/− 0.062	0.691 +/− 0.043
*SVM-linear*	0.846 +/− 0.026	0.7685 +/− 0.033	0.954 +/− 0.027	0.583 +/− 0.039	0.609 +/− 0.071
*SVM-radial*	0.737 +/− 0.007	0.5475 +/− 0.0075	1 +/− 0	0.095 +/− 0.015	0.262 +/− 0.024
*SVM-sigmoid*	0.647 +/− 0.047	0.5365 +/− 0.063	0.8 +/− 0.038	0.273 +/− 0.088	0.079 +/− 0.122
*LDA*	0.68 +/− 0.041	0.676 +/− 0.053	0.687 +/− 0.049	0.665 +/− 0.057	0.325 +/− 0.074
*CART*	0.804 +/− 0.016	0.752 +/− 0.044	0.876 +/− 0.028	0.628 +/− 0.06	0.517 +/− 0.043
*NN*	0.795 +/− 0.028	0.7485 +/− 0.04	0.86 +/− 0.026	0.637 +/− 0.054	0.501 +/− 0.065
***Fitting (training set, n*** = ***126)***					
***Method***	***Acc***	***Acc***_***b***_	***Sp***	***Se***	***MCC***
*RF*	0.99 +/− 0.005	0.9825 +/− 0.009	1 +/− 0	0.965 +/− 0.017	0.975 +/− 0.012
*SVM-linear*	0.934 +/− 0.033	0.895 +/− 0.0565	0.989 +/− 0.01	0.801 +/− 0.103	0.839 +/− 0.083
*SVM-radial*	0.98 +/− 0.005	0.966 +/− 0.0095	1 +/− 0	0.932 +/− 0.019	0.953 +/− 0.013
*SVM-sigmoid*	0.622 +/− 0.055	0.517 +/− 0.089	0.764 +/− 0.075	0.27 +/− 0.103	0.043 +/− 0.113
*LDA*	0.997 +/− 0.004	0.9955 +/− 0.007	1 +/− 0	0.991 +/− 0.013	0.994 +/− 0.009
*CART*	0.104 +/− 0.014	0.1335 +/− 0.057	0.064 +/− 0.031	0.203 +/− 0.082	−0.748 +/− 0.034
*NN*	0.958 +/− 0.028	0.9485 +/− 0.036	0.973 +/− 0.022	0.924 +/− 0.05	0.899 +/− 0.069
***External validation (test set, n*** = ***22)***					
***Method***	***Acc***	***Acc***_***b***_	***Sp***	***Se***	***MCC***
*RF*	0.868 +/− 0.04	0.7995 +/− 0.121	0.949 +/− 0.055	0.65 +/− 0.186	0.672 +/− 0.092
*SVM-linear*	0.839 +/− 0.092	0.7705 +/− 0.1175	0.932 +/− 0.101	0.609 +/− 0.134	0.621 +/− 0.172
*SVM-radial*	0.727 +/− 0.076	0.529 +/− 0.0355	1 +/− 0	0.058 +/− 0.071	0.126 +/− 0.156
*SVM-sigmoid*	0.587 +/− 0.1	0.5355 +/− 0.162	0.701 +/− 0.094	0.37 +/− 0.23	0.038 +/− 0.219
*LDA*	0.64 +/− 0.114	0.625 +/− 0.168	0.664 +/− 0.143	0.586 +/− 0.192	0.235 +/− 0.217
*CART*	0.229 +/− 0.077	0.27 +/− 0.155	0.191 +/− 0.115	0.349 +/− 0.195	−0.46 +/− 0.186
*NN*	0.845 +/− 0.071	0.808 +/− 0.1605	0.882 +/− 0.065	0.734 +/− 0.256	0.61 +/− 0.222

Chemicals active under any cell culture condition were considered as actives for each color channel. Each model building process was repeated 10 times with distinct data segregation and inactive under sampling from the entire Tox21 dataset, and the mean (M) and the standard deviation (SD) of each performance criterion are reported, Acc: accuracy, Acc_b_: balanced accuracy, Sp: specificity, Se: sensitivity and MCC: Matthew Coefficient Correlation, see methods.

Supplemental Tables S4–S7 show all model performances for any combination of cell culture condition and color channel. On the blue channel QSAR models had weaker performance predicting autofluorescence under cell-based conditions with a cross validation MCC of around 0.44, due to the set of 111 chemicals without concurrent blue channel activity on other cell culture conditions (Fig. 7). For the other culture-specific QSAR models, the performances are close to the color specific QSAR models’ performance presented in Table 4, due to a high degree of consistency among the red and green actives within each channel across conditions.

### QSAR model descriptors

**Figure 9:**
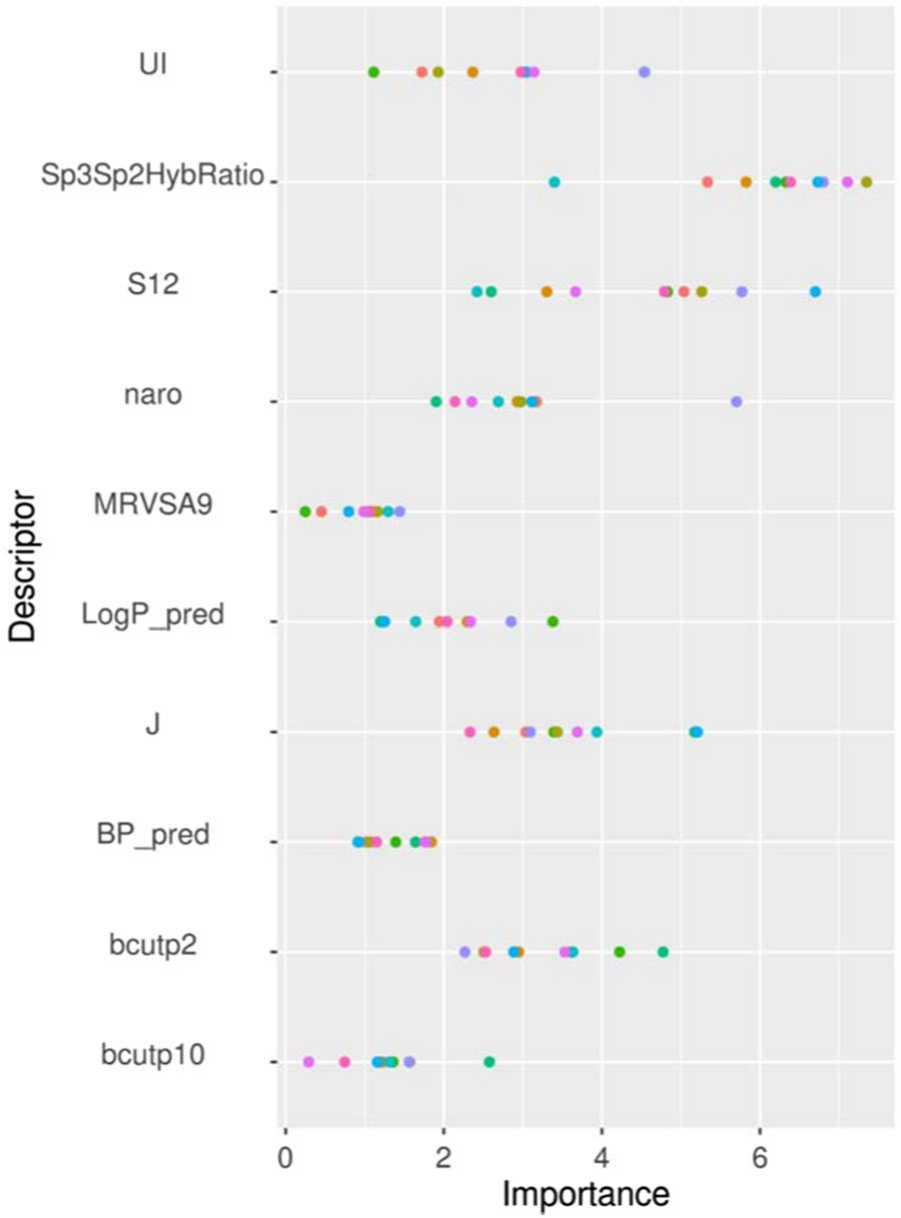
Variable importance plots for the top 10 descriptors from the random forest QSAR models predicting luciferase inhibition. Ten values for each descriptor are reported, corresponding to each of the ten models developed with different data set segregations. Descriptors are defined in Supporting Information Table S8.

**Figure 10:**
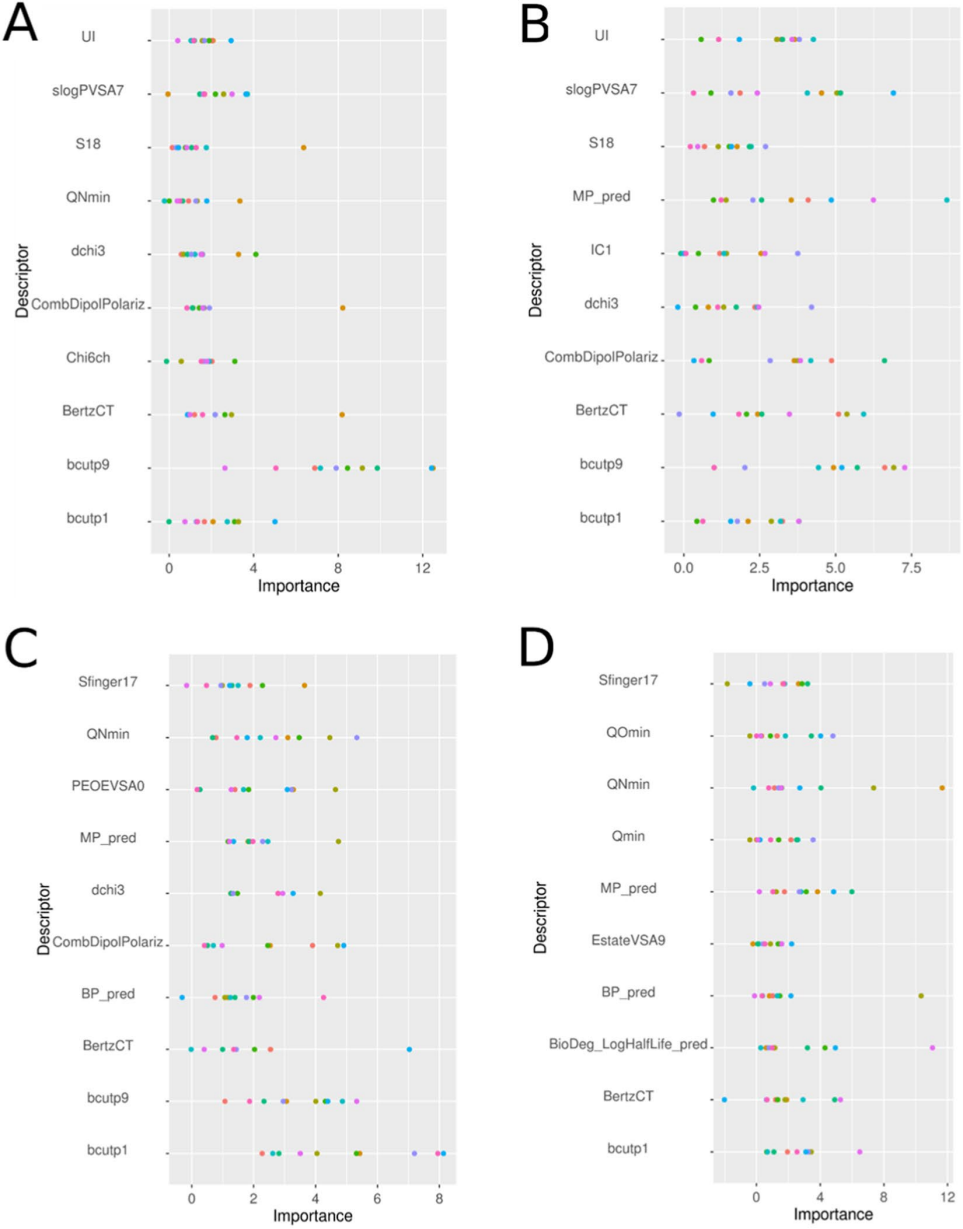
Variable importance plots for the top 10 descriptors from the random forest QSAR models predicting autofluorescence in: (**A**) any color channel, (**B**) the blue channel, (**C**) the green channel and (**D**) the red channel. The importance of each descriptor is normalized, and ten values for each descriptor are reported corresponding to each of the ten models developed with different data set segregations. Descriptors are defined in Supporting Information Table S8.

The variable importance scores of the RF QSAR model descriptors are presented in Fig. 9 for luciferase and Fig. 10 for autofluorescence assays and are summarized in Supporting Information Table S8. For all models, the most important descriptors include at least one feature characterizing the polarizability of the chemical (CombDipolPolariz or bcutp) and one of the following physicochemical properties (UI, logP_pred, BP_pred, MP_pred or BioDegHL_pred). Specifically, for luciferase inhibition the most important descriptors characterize the ratio between unsaturated/saturated bonds (Sp3Sp2HybRatio), followed by the E-state of a methyl connected to an aromatic (S12). On average, active chemicals have a lower ratio between unsaturated/saturated bonds (0.21 for actives and 0.48 for inactives) and have an energy state more influenced by methyl group connected to an aromatic than inactive (S12) (11.15 for actives and 5.74 for inactives, Supporting Information Table S9). For autofluorescence QSARs, unsaturated index (UI) descriptors, which characterize the ratio between unsaturated and saturated bonds in a chemical, were found in almost every model as an important descriptor. The unsaturated index is higher for active chemicals (~3.5) than inactive (~2.6) due to the fact that active chemical are mostly enriched in aromatic groups. Burden descriptors (bcutp), which characterize mass by atom type and bond, are also present in every model and burden descriptors are higher for active chemicals than inactive. The red channel model includes three descriptors characterizing the charges (QNmin, Qmin and QOmin) and active chemicals are less charged that inactives. The QSAR models built individually on the blue and green color channels shared six of the top 10 important descriptors, and the blue channel and luciferase inhibition models shared four important descriptors.

## Discussion

Out of the large, diverse chemical set represented by the Tox21 library, approximately 10% of chemicals demonstrated some degree of assay interference potential depending on the endpoint (luciferase inhibition or autofluorescence via blue, green, and/or red channels). These chemicals cover a wide range of commercial uses and regulatory lists of concern, with varying degrees of associated environmental occurrence and toxicity data^42^. In most cases, data generated under the Tox21 program represent the bulk of bioactivity information available for a particular chemical, highlighting the importance of identifying false signals to allow for true characterization of chemical-target activity driven by biological and toxicological perturbations.

Luciferase inhibition was the most prevalent interference activity observed among the Tox21 chemicals (Table 1). This assay relies on loss of signal, which would potentially be interpreted as a false positive for the antagonist or inhibitory mode of a luciferase-based reporter gene assay measuring biological activity. For autofluorescence, there were many more chemicals active in the blue channel (~3%) than in the green (~1%) or red (~0.5%) channels. These results are in agreement with other studies showing that around 5% of the chemicals in a diverse library can fluoresce in the blue spectrum versus less than 1% for the other parts of the spectrum^10,46^. Interestingly, the activity density plots for all three color wavelengths demonstrated bimodal potency distributions, with two peaks around 10 and 40 µM, and the majority of AC_50_ values falling below 50 µM (Fig. 4).

Multiple supervised and unsupervised machine learning and clustering approaches were harnessed to analyze the interference activity data and characterize structural patterns associated with luciferase inhibition or autofluorescence among different wavelengths. For all of the clusters discussed in the SOM for luciferase inhibition (Fig. 5A), the structural scaffolds found were consistent with those that have been previously characterized in the literature as Fluc inhibitors^9,11^. When examining the SOM for autofluorescence (Fig. 5B), it is clear that a high number of rings in a scaffold is associated with fluorescence activity, as discussed in Su *et al*.^14^, where the authors dictated that the presence of more than six rings in a chemical fulfilled criteria to be a strong fluorophore. This observation was also consistent when examining chemicals that were strongly interferent, i.e. active across all wavelengths and culture conditions, where a high number of rings (and the presence of at least one oxygen) increases the ability to absorb light. Further known and novel associations were identified between various structural scaffolds and autofluorescence activity (Figs. 4A–C and 5B), and channel/condition-specific potency/activity (Fig. 8A–C), prompting the development of structure-based prediction models.

QSAR models were developed using four machine learning algorithms. Across models, CART and SVM nonlinear were not able to discriminate active and inactive chemicals, where SVM models tended to overfit the training set with accuracies close to 100% and CART models poorly predicted the test sets (negative MCC). The lack of performance of these models is explained by the unbalanced set and the difficulty to linearly separate active and inactive chemicals due to the large diversity of inactive chemical (see PCA plot, Supporting Information Fig. S6). Overall, RF models exhibited the best predictive performance, as discussed below.

The QSAR models for luciferase inhibition demonstrated high predictive performance, with ~83% external test set accuracy values (Table 2) and balanced accuracy equal to 76%. For each color channel in the autofluorescence dataset, there were varying degrees of chemical activity in specific cell culture conditions, but the largest number of active chemicals was always found in the intersection among all conditions (Fig. 7), representing those compounds that could be considered “strongly interferent” for a particular wavelength. These strong actives were used as “true positives” in the machine learning approaches applied to build models for general autofluorescence, which demonstrated over 82% accuracy on external validation sets (Table 3) with balanced accuracy of 77%. There was no significant difference between percent actives in individual cell culture conditions within or across wavelengths, indicating that one particular cell line or media choice did not play a large role in driving autofluorescent interference. However, structure-based analysis did reveal clusters of active chemicals specific to certain cell culture conditions, and the QSAR models that were built for individual channel/culture combinations demonstrated high performance (76–92% external set accuracy, Supporting Information Table S4–S7). In the case of the red channel models, this is due to the high degree of consistency where active chemicals were generally active across culture conditions (Fig. 8C), meaning that the individual culture condition models are essentially equivalent to the red channel model overall. For the blue channel models there were higher numbers of active chemicals in individual culture conditions (Fig. 8A), and the respective structure-based prediction models therefore provide unique insight.

As would be expected based on the number of chemicals and patterns of activity across culture conditions within each channel, the highest performing color-specific model was on the red channel, with 86–87% external set accuracies and 80% balanced accuracy, followed by the green channel with 85–86% external set accuracy and 81% balanced accuracy. Performances were lower on the blue channel with external set accuracies of 77–78% and a balanced accuracy equal to 68% due to the larger number of diverse chemical structures with disparate activity across cell culture conditions. For every model, loss of performance can be explained by low sensitivity, i.e. the difficulty in predicting active chemicals. This phenomenon is explained by the structural diversity of the large inactive set, and the coverage of the active set by the inactive set, where inactive chemicals share many structural features with active chemicals as shown in Supporting Information Fig. S6 by the high density of the PCA map. The lack of purity of clusters in the SOM clustering (Figs. 5 and 7) below 70% actives also shows the proximity between active and inactive chemicals.

The developed QSAR models were applied to predict interference for 4632 small molecules included in the DrugBank database with structures available on the EPA comptox chemical dashboard (https://comptox.epa.gov/dashboard/chemical_lists/DRUGBANK). From this list of chemicals, 1495 were found in the Tox21 chemical library, and performances on this subset of chemicals with assay data were found to be consistent with the performances on the test set with an accuracy of 0.94 and a MCC equal to 0.63 for luciferase models, and an accuracy of 0.88 and a MCC equal to 0.33 for autofluorescence models (merging conditions, cell lines and wave lengths). The interference probability distribution (Supplemental Fig. S7 panel A), shows that most chemicals were predicted not to interfere with assay technologies, while the distribution of those that were predicted as interferent compounds (Supplemental Fig. S7 panel B) was consistent with the Tox21 chemical library (Fig. 7). For example, the models identified Lymecycline (CAS: 992–21–2) as an interferent chemical for all of the autofluorescence models in the blue and green. Lymecycline belongs to the tetracycline family, which are known to absorb the light at low wave lengths (Conover *et al*. 1953) which is consistent with interference in low absorbance color ranges (blue and green). The QSAR predictions for this list of drugs/small molecules have been included in supporting information.

The application of RF models to predict luciferase inhibition and autofluorescence allows for interrogation of important structural features and physicochemical properties driving the model performance (variable importance, Fig. 9). Over 50% of the important descriptors found were dependent or connected to aromatic properties, confirming that aromatic rings play an important role in the absorption/emission of light and, as previously discussed, aromatic rings are known to influence fluorescence chemical properties^14^. When examining the reference chemicals for each channel, this is apparent. The scaffold of triamterene is composed of a pteridine ramified with three primary amines and one benzene, and includes only nitrogen and carbon as heavy atoms. Fluorescein is composed of a dioxaspirane ramified with one benzene and two phenol groups, and includes only carbon and oxygen. Finally, rose bengal includes a xanthene group ramified with four iodine atoms, two ketone and one tetrachlorinebenzoic acid group, and more diverse atom types than the Triamterene and the fluorescein, with chlorine and iodine atoms. The red channel model includes three descriptors characterizing the charges, which can be explained by the more diverse composition of heteroatoms in active chemicals on the red channel as shown for example with rose bengal, chemical 4 in Fig. 4, including iodine and chlorine. It is also worth noting that the blue channel model and the luciferase inhibition model shared four of the most important descriptors, which can be explained by the number of chemicals (26) active in both assays (Supporting Information Fig. S3).

The work discussed here represents one of the largest screening efforts to date specifically intended to identify and characterize chemical-assay interference via luciferase inhibition and autofluorescence, and to interrogate the influence of cell types and culture conditions. The resulting predictive models (https://sandbox.ntp.niehs.nih.gov/interferences) can be used to predict interference potential of new chemicals, and to provide insight into structural features that may influence activity and inform molecular design and assay selection.

## Supplementary information

Supplementary information.

Supplementary Dataset 1.

Supplementary Dataset 2.

Supplementary Dataset 3.

Supplementary Dataset 4.
