# Supplementary material for: High-Throughput Screening to Predict Chemical-Assay Interference
Source: Sci Rep. 2020 Mar 4;10:3986. doi: 10.1038/s41598-020-60747-3 (PMC7055224; doi:10.1038/s41598-020-60747-3)
Supplement: Supplementary file 1 — Supplementary information. [file 41598_2020_60747_MOESM1_ESM.docx]

**Supporting information**

High-Throughput Screening to Predict Chemical-Assay Interference

A Borrel^1^, R Huang^2^, S Sakamuru^2^, M Xia^2^, A Simeonov^2^, K Mansouri^3^, K Houck^4^, R Judson^4^, N Kleinstreuer^1,5*^

^1^NIH/NIEHS/DIR/BCBB, RTP, NC, United States; ^2^NIH/NCATS, Bethesda, MD, United States; ^3^Integrated Laboratory Systems, RTP, NC, United States; ^4^EPA/ORD/NCCT, RTP, NC, United States; ^5^NIH/NIEHS/DNTP/NICEATM, RTP, NC, United States

*Correspondence to nicole.kleinstreuer@nih.gov

**Data Tables (Excel files)**

1. Selected descriptors table for the Tox21 chemical library (descriptors.xlsx)
2. Table of AC50/IC50 for each assays/cell culture/condition after filtering (AC50-IC50_all.xlsx)
3. Tox21 chemical library classification using chemical consumer product classes and the TSCA, approved drug classes (chemical_classes.xlsx)
4. Prediction results of the 17 developed QSAR interference models on drug chemicals available in EPA chemical dashboard (<https://comptox.epa.gov/dashboard/chemical_lists/DRUGBANK>) (drugbank_prediction.xlsx).

***Table S1****: Curve classification criteria, adapted from* ^1,2^*. SD is the standard deviation of sample activities at the lowest tested concentration and DMSO control well values .*

| Curve class | Description | Efficacy | r^2^ | Asymptotes | Inflexion |
| --- | --- | --- | --- | --- | --- |
| (-) 1.1 | Complete response | > 6SD | r2 ≥ 0.9 | 2 | Yes |
| (-) 1.2 |  | ≤ 6SD; > 3SD | r2 ≤ 0.9 |  |  |
| (-) 1.3 |  | > 6SD |  |  |  |
| (-) 1.4 |  | ≤ 6SD; > 3SD |  |  |  |
| (-) 2.1 | Incomplete curve | > 6SD | r2 ≥ 0.9 | 1 |  |
| (-) 2.2 |  | ≤ 6SD; > 3SD | r2 ≤ 0.9 |  |  |
| (-) 2.3 |  | > 6SD |  |  |  |
| (-) 2.4 |  | ≤ 6SD; > 3SD |  |  |  |
| (-) 3 | Single point activity | > 3SD | NA | 0 | No |
| (-) 4 | Inactive | ≤ 3SD |  | NA |  |
| (-) 5 | Inconclusive | NA |  |  | NA |

1. Huang, R. *et al.* Chemical genomics profiling of environmental chemical modulation of human nuclear receptors. *Environ. Health Perspect.* **119,** 1142–1148 (2011).

2. Inglese, J. *et al.* Quantitative high-throughput screening: A titration-based approach that efficiently identifies biological activities in large chemical libraries. *Proc. Natl. Acad. Sci.* **103,** 11473–11478 (2006).

**Table S2**: Assays results summary: count of active chemicals computed based on the 8,305 chemicals tested from Tox21 after the different filters applied sequentially.

|  |  |  | filter applied sequentially, count of active | | | |
| --- | --- | --- | --- | --- | --- | --- |
|  | Cell culture | Type of AC50/IC50 | initial | curve | effect | burst |
| Luciferase |  | Luciferase | 5413 | 1330 | 552 | - |
| Auto-fluorescence | HepG2 | Cell based blue | 2657 | 302 | 236 | 216 |
|  |  | Cell based green | 4216 | 110 | 86 | 80 |
|  |  | Cell based red | 3752 | 68 | 39 | 39 |
|  |  | Cell free blue | 3176 | 283 | 226 | 209 |
|  |  | Cell free green | 4535 | 106 | 89 | 80 |
|  |  | Cell free red | 5592 | 62 | 37 | 37 |
|  | HEK293 | Cell based blue | 2852 | 331 | 231 | 210 |
|  |  | Cell based green | 1393 | 69 | 59 | 56 |
|  |  | Cell based red | 4295 | 41 | 33 | 33 |
|  |  | Cell free blue | 3159 | 296 | 245 | 224 |
|  |  | Cell free green | 1626 | 69 | 48 | 44 |
|  |  | Cell free red | 4560 | 51 | 34 | 34 |


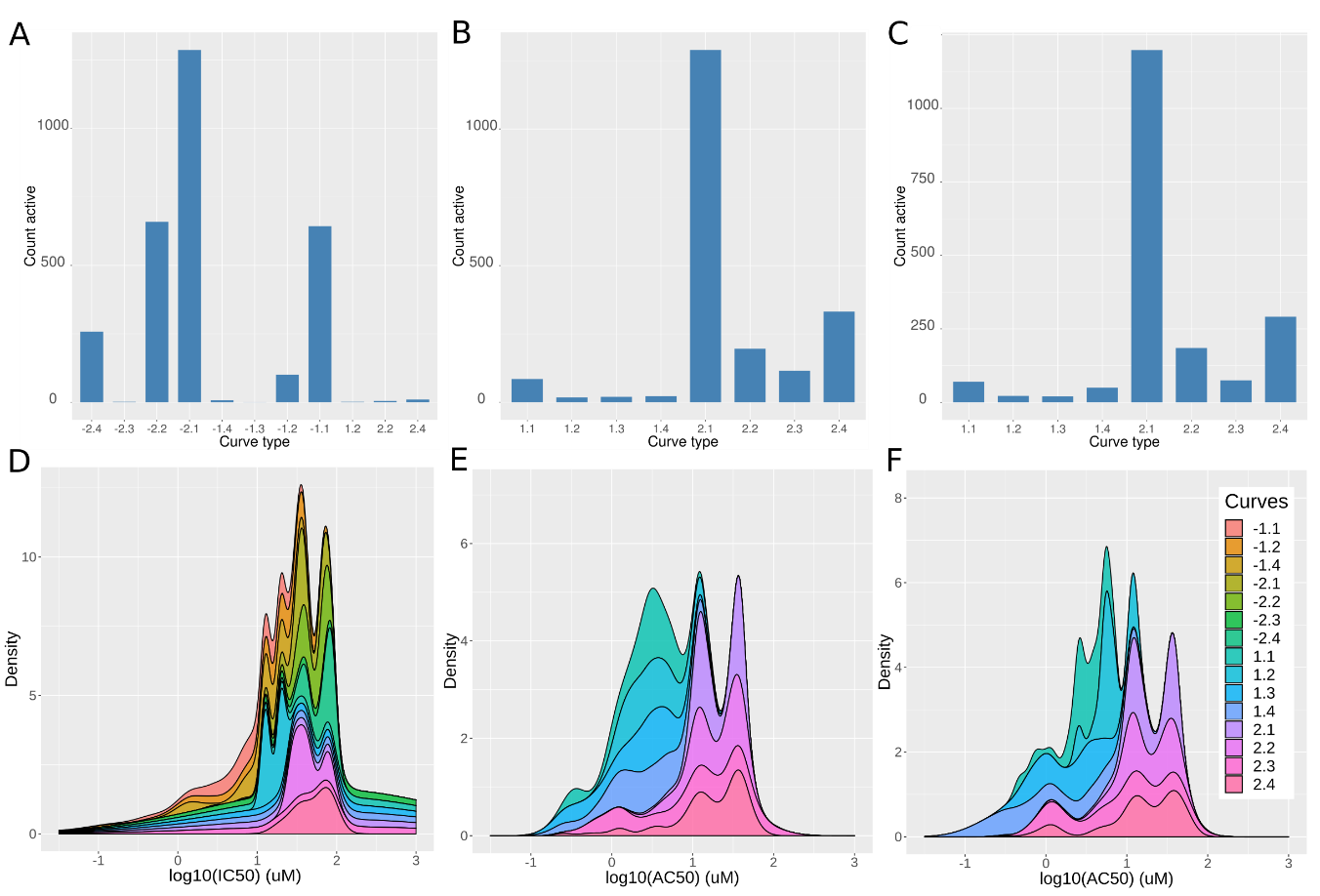


**Figure S1**: Distribution of active chemicals and response curve classes for A. luciferase inhibition assay, and autofluorescence assays using B. HepG2 cell culture conditions and C. Hek293 cell culture conditions. Panels D-F represent the distribution of IC50/ AC50 with the response curves density type for D. luciferase assay, and for autofluorescence assays using E. HepG2 and F. Hek293 cell culture conditions.


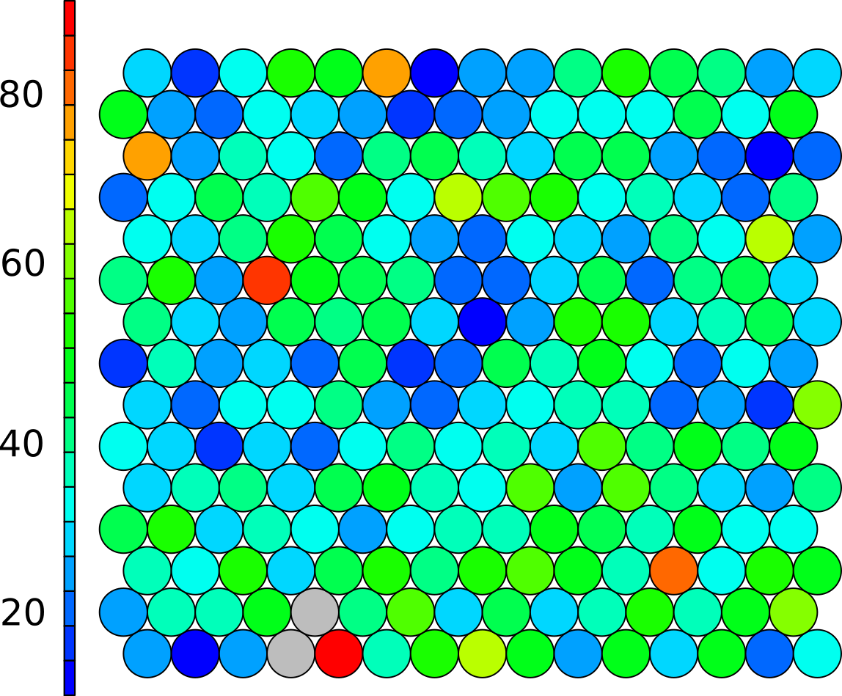


**Figure S2**: Structure base SOM on 8,065 chemicals including in the Tox21, color represented the count of chemical by cluster (scale on left). 225 clusters are included in total.


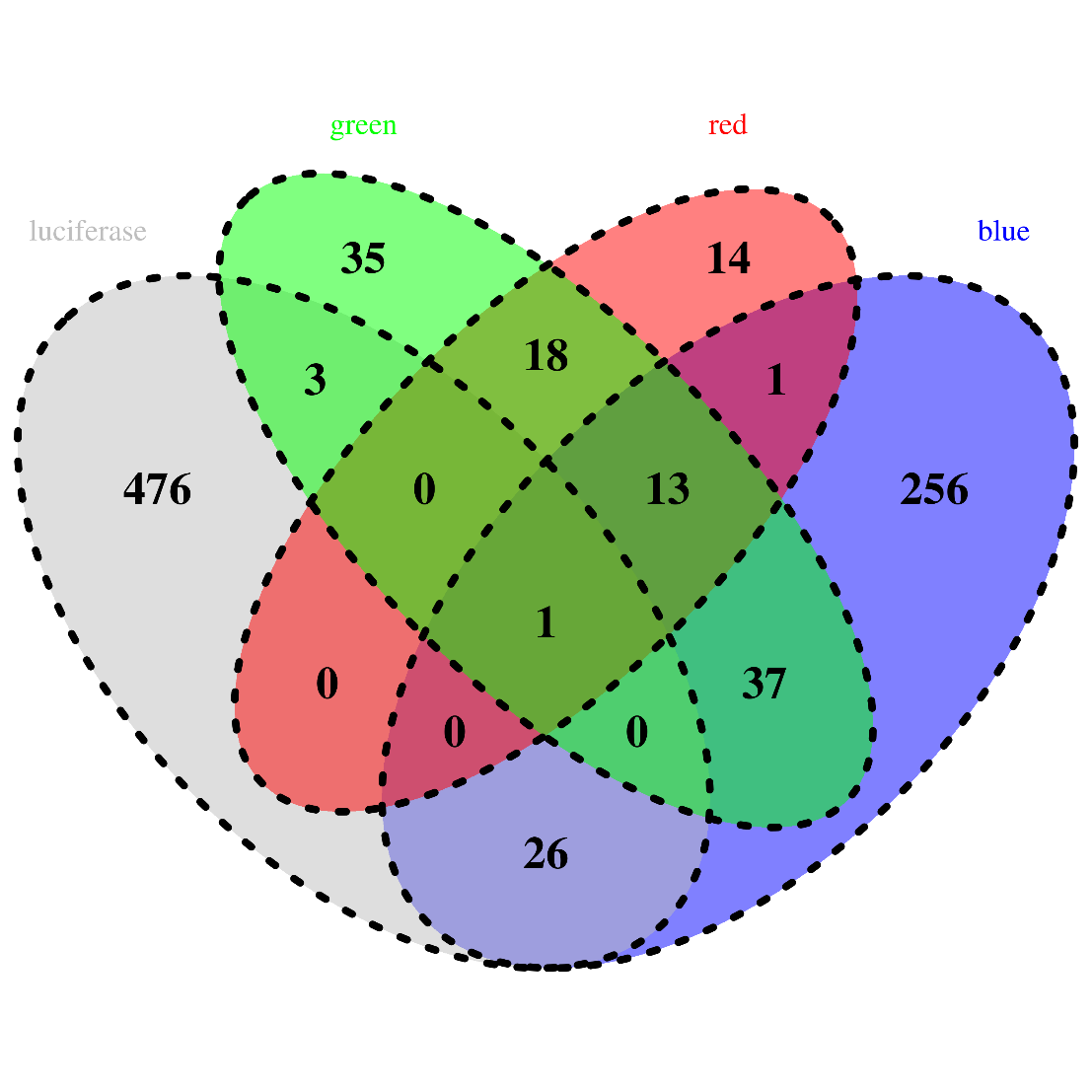


**Figure S3**: Venn diagram of active chemicals on luciferase and autofluorescence assays for channels blue, green and red.

**Table S3:** active chemicals on all autofluorescence assays for any color channel and cell culture conditions.


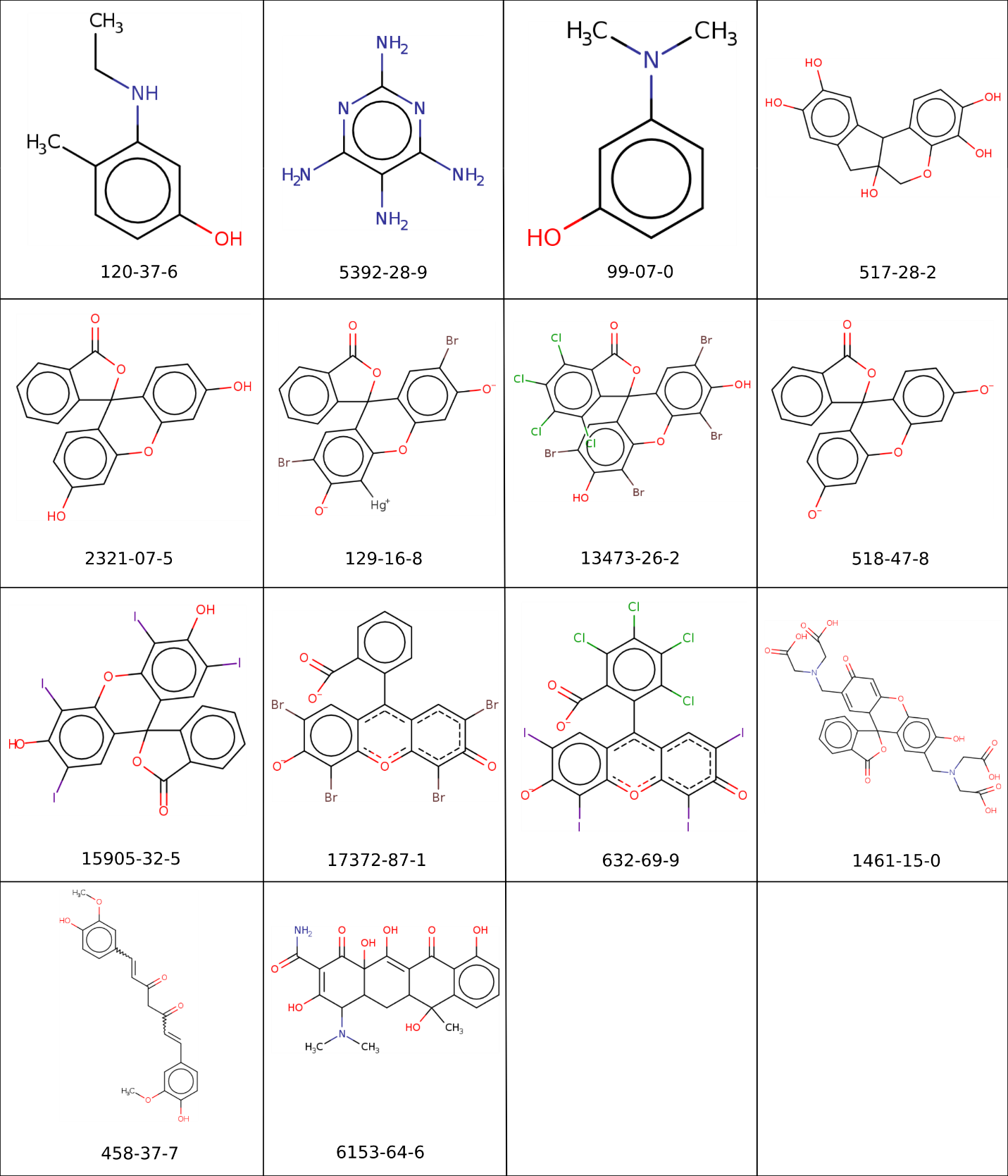


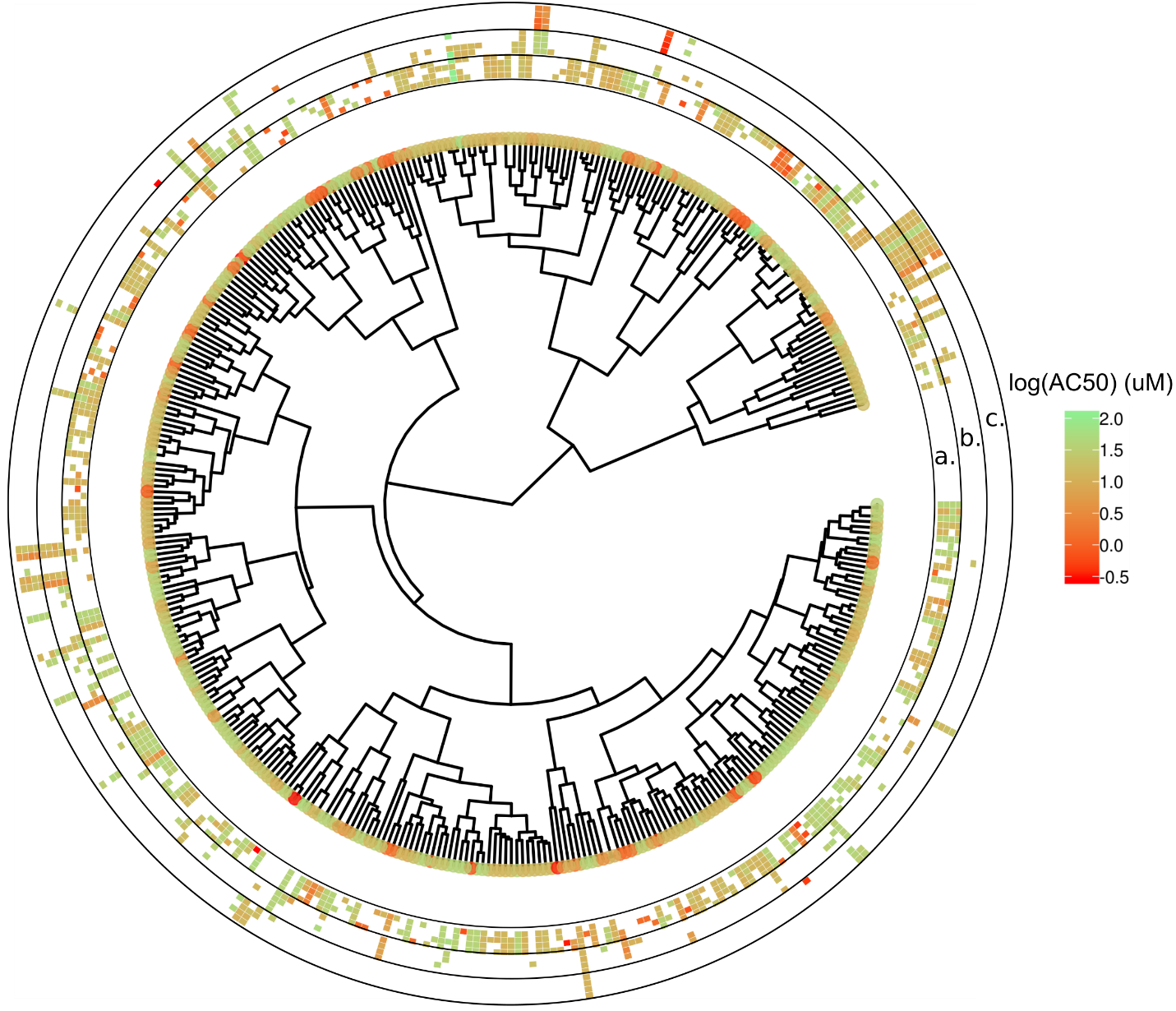


**Figure S4**: Hierarchical clustering of active chemicals on autofluorescence assays for a. blue, b. green and c. red channel across all cell culture conditions. Hierarchical clustering is realized using a Euclidean distance computed from a set of 165 non-redundant molecular descriptors and using Ward segregation, see methods. Potency in terms of AC50 for each chemical is represented using a color scale from red to green. Chemical activity in each channel is represented by four AC50s from inner to outer circle: HepG2 cell-based condition, HepG2 cell-free condition, HEK-293 cell-based condition and HEK-293 cell-free condition.


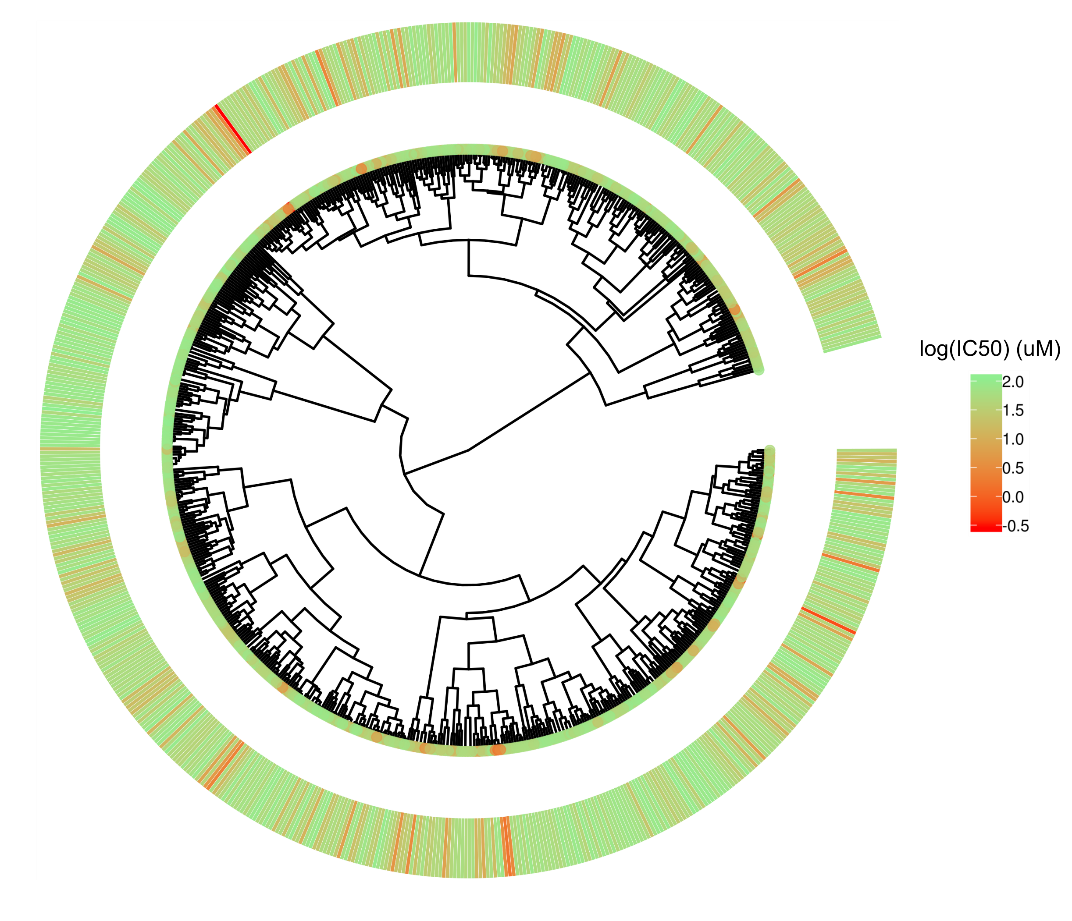


**Figure S5**: Hierarchical clustering of active chemicals in luciferase inhibition assay. Hierarchical clustering is realized using a Euclidean distance computed from a set of non-redundant molecular descriptor and using Ward segregation, see methods. AC50 for each


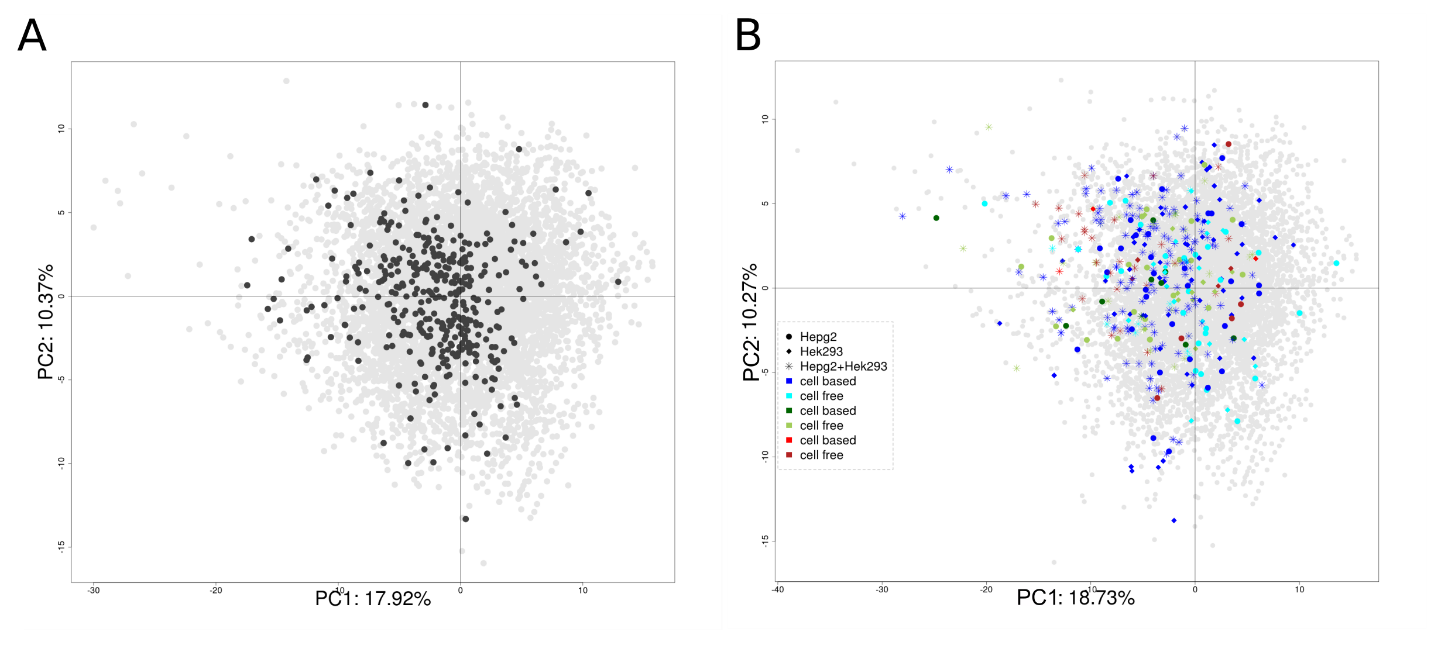


**Figure S6:** Principal component analysis of the Tox21 chemical library computed using the set of 165 selected descriptors. The first component explains 19.88% of the descriptor variability and the second 9.85%. Chemicals are colored based on their activity in black for active chemicals on luciferase assays (A) and in blue, green and red for active chemicals on autofluorescence on blue, green and red channel respectively (B). Chemicals active only in HepG2 cell culture conditions are represented by a circle, only in HEK293 cell culture conditions by a diamond, and in both by a star.


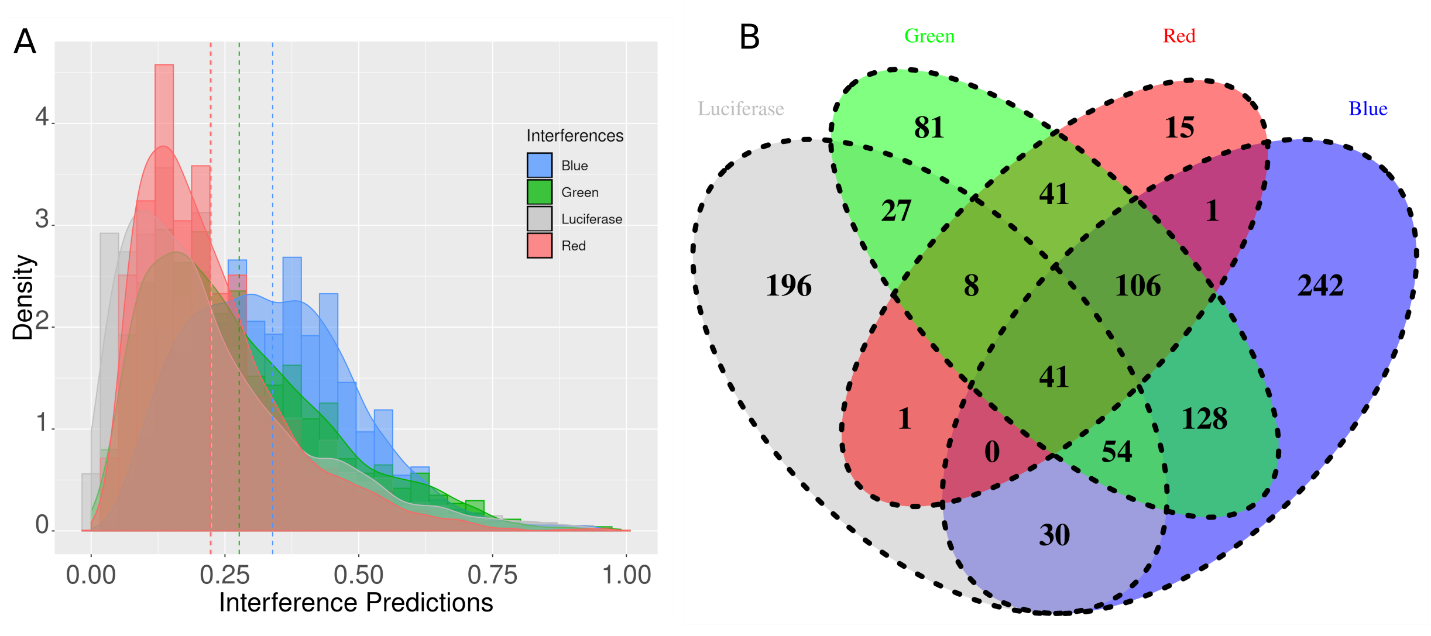


**Figure S7**: Prediction interferences results for drug chemicals available in the DSSTOX database, A-interference probability distribution for predicted chemicals using luciferase and blue, green, red autofluorescence interference models. B- Venn diagram for predicted interference chemicals (probability > 0.5) using luciferase and blue, green, red autofluorescence models.

**Table S4**: Performance of QSAR classification models for autofluorescence activity specific to wavelength and HepG2 cell-based assays. Each model building process was repeated 10 times with distinct data segregation and inactive undersampling from the entire Tox21 dataset, and the mean (M) and the standard deviation (SD) of each performance criterion are reported, Acc: accuracy, Sp: specificity, Se: sensitivity and MCC Matthew Coefficient Correlation, see methods.

| **Autofluorescence assays (HepG2 culture cell)** | | | | | | | | |
| --- | --- | --- | --- | --- | --- | --- | --- | --- |
| **Blue – cell based AC50** | | | | | | | | |
|  | ***10-fold cross-validation (full set, n=536)*** | | | | | | | |
| ***Method*** | ***M Acc*** | ***SD Acc*** | ***M Sp*** | ***SD Sp*** | ***M Se*** | ***SD Se*** | ***M MCC*** | ***SD MCC*** |
| ***LDA*** | 0.781 | 0.014 | 0.856 | 0.016 | 0.616 | 0.025 | 0.482 | 0.032 |
| ***SVM-linear*** | 0.797 | 0.01 | 0.918 | 0.014 | 0.532 | 0.03 | 0.501 | 0.026 |
| ***NN*** | 0.758 | 0.014 | 0.839 | 0.017 | 0.579 | 0.045 | 0.426 | 0.037 |
| ***SVM-radial*** | 0.711 | 0.004 | 0.999 | 0.001 | 0.077 | 0.01 | 0.225 | 0.018 |
| ***RF*** | 0.81 | 0.009 | 0.918 | 0.012 | 0.572 | 0.032 | 0.536 | 0.025 |
| ***SVM-sigmoid*** | 0.655 | 0.015 | 0.796 | 0.018 | 0.344 | 0.015 | 0.15 | 0.031 |
| ***CART*** | 0.758 | 0.017 | 0.85 | 0.012 | 0.557 | 0.036 | 0.421 | 0.045 |
|  | ***Fitting (training set, n=541)*** | | | | | | | |
| ***Method*** | ***M Acc*** | ***SD Acc*** | ***M Sp*** | ***SD Sp*** | ***M Se*** | ***SD Se*** | ***M MCC*** | ***SD MCC*** |
| ***LDA*** | 0.887 | 0.009 | 0.943 | 0.007 | 0.765 | 0.033 | 0.732 | 0.026 |
| ***SVM-linear*** | 0.835 | 0.009 | 0.94 | 0.013 | 0.603 | 0.034 | 0.6 | 0.025 |
| ***NN*** | 0.851 | 0.018 | 0.903 | 0.028 | 0.735 | 0.055 | 0.65 | 0.044 |
| ***SVM-radial*** | 0.973 | 0.005 | 1 | 0.001 | 0.915 | 0.015 | 0.938 | 0.011 |
| ***RF*** | 0.998 | 0.002 | 0.999 | 0.001 | 0.994 | 0.006 | 0.994 | 0.005 |
| ***SVM-sigmoid*** | 0.623 | 0.019 | 0.762 | 0.027 | 0.316 | 0.02 | 0.083 | 0.036 |
| ***CART*** | 0.117 | 0.008 | 0.064 | 0.017 | 0.234 | 0.044 | -0.723 | 0.021 |
|  | ***External validation (test set, n=95)*** | | | | | | | |
| ***Method*** | ***M Acc*** | ***SD Acc*** | ***M Sp*** | ***SD Sp*** | ***M Se*** | ***SD Se*** | ***M MCC*** | ***SD MCC*** |
| ***LDA*** | 0.804 | 0.046 | 0.873 | 0.038 | 0.652 | 0.083 | 0.536 | 0.101 |
| ***SVM-linear*** | 0.801 | 0.034 | 0.908 | 0.03 | 0.57 | 0.085 | 0.515 | 0.076 |
| ***NN*** | 0.783 | 0.026 | 0.842 | 0.043 | 0.649 | 0.069 | 0.493 | 0.048 |
| ***SVM-radial*** | 0.714 | 0.041 | 0.997 | 0.006 | 0.091 | 0.028 | 0.237 | 0.039 |
| ***RF*** | 0.812 | 0.028 | 0.906 | 0.031 | 0.608 | 0.06 | 0.547 | 0.051 |
| ***SVM-sigmoid*** | 0.632 | 0.038 | 0.766 | 0.048 | 0.338 | 0.085 | 0.107 | 0.075 |
| ***CART*** | 0.221 | 0.043 | 0.148 | 0.033 | 0.383 | 0.111 | -0.474 | 0.108 |
| **Green – cell based AC50** | | | | | | | | |
|  | ***10-fold cross-validation (full set, n=166)*** | | | | | | | |
| ***Method*** | ***M Acc*** | ***SD Acc*** | ***M Sp*** | ***SD Sp*** | ***M Se*** | ***SD Se*** | ***M MCC*** | ***SD MCC*** |
| ***LDA*** | 0.77 | 0.02 | 0.79 | 0.016 | 0.719 | 0.042 | 0.482 | 0.047 |
| ***SVM-linear*** | 0.826 | 0.012 | 0.942 | 0.017 | 0.539 | 0.046 | 0.55 | 0.033 |
| ***NN*** | 0.799 | 0.032 | 0.85 | 0.022 | 0.672 | 0.062 | 0.517 | 0.079 |
| ***SVM-radial*** | 0.728 | 0.003 | 1 | 0 | 0.058 | 0 | 0.205 | 0 |
| ***RF*** | 0.861 | 0.011 | 0.929 | 0.01 | 0.693 | 0.041 | 0.651 | 0.029 |
| ***SVM-sigmoid*** | 0.703 | 0.039 | 0.832 | 0.031 | 0.386 | 0.067 | 0.234 | 0.102 |
| ***CART*** | 0.785 | 0.022 | 0.861 | 0.017 | 0.6 | 0.085 | 0.469 | 0.065 |
|  | ***Fitting (training set, n=141)*** | | | | | | | |
| ***Method*** | ***M Acc*** | ***SD Acc*** | ***M Sp*** | ***SD Sp*** | ***M Se*** | ***SD Se*** | ***M MCC*** | ***SD MCC*** |
| ***LDA*** | 1 | 0 | 1 | 0 | 1 | 0 | 1 | 0 |
| ***SVM-linear*** | 0.883 | 0.027 | 0.975 | 0.007 | 0.649 | 0.101 | 0.699 | 0.079 |
| ***NN*** | 0.907 | 0.061 | 0.941 | 0.027 | 0.828 | 0.213 | 0.773 | 0.158 |
| ***SVM-radial*** | 0.982 | 0.006 | 1 | 0 | 0.937 | 0.024 | 0.956 | 0.016 |
| ***RF*** | 0.998 | 0.002 | 1 | 0 | 0.993 | 0.008 | 0.995 | 0.006 |
| ***SVM-sigmoid*** | 0.661 | 0.04 | 0.8 | 0.037 | 0.315 | 0.102 | 0.122 | 0.125 |
| ***CART*** | 0.089 | 0.021 | 0.056 | 0.014 | 0.171 | 0.087 | -0.78 | 0.061 |
|  | ***External validation (test set, n=25)*** | | | | | | | |
| ***Method*** | ***M Acc*** | ***SD Acc*** | ***M Sp*** | ***SD Sp*** | ***M Se*** | ***SD Se*** | ***M MCC*** | ***SD MCC*** |
| ***LDA*** | 0.655 | 0.042 | 0.658 | 0.085 | 0.628 | 0.121 | 0.267 | 0.092 |
| ***SVM-linear*** | 0.795 | 0.08 | 0.896 | 0.045 | 0.581 | 0.142 | 0.504 | 0.173 |
| ***NN*** | 0.777 | 0.069 | 0.827 | 0.072 | 0.67 | 0.139 | 0.487 | 0.16 |
| ***SVM-radial*** | 0.695 | 0.066 | 1 | 0 | 0 | 0 | 0 | 0 |
| ***RF*** | 0.868 | 0.057 | 0.89 | 0.056 | 0.834 | 0.103 | 0.702 | 0.125 |
| ***SVM-sigmoid*** | 0.656 | 0.062 | 0.788 | 0.089 | 0.389 | 0.155 | 0.178 | 0.107 |
| ***CART*** | 0.194 | 0.044 | 0.14 | 0.067 | 0.309 | 0.043 | -0.547 | 0.108 |
| **Red – cell based AC50** | | | | | | | | |
|  | ***10-fold cross-validation (full set, n=95)*** | | | | | | | |
| ***Method*** | ***M Acc*** | ***SD Acc*** | ***M Sp*** | ***SD Sp*** | ***M Se*** | ***SD Se*** | ***M MCC*** | ***SD MCC*** |
| ***LDA*** | 0.723 | 0.029 | 0.737 | 0.018 | 0.688 | 0.068 | 0.392 | 0.071 |
| ***SVM-linear*** | 0.84 | 0.004 | 0.948 | 0.014 | 0.563 | 0.026 | 0.579 | 0.008 |
| ***NN*** | 0.825 | 0.02 | 0.895 | 0.029 | 0.646 | 0.039 | 0.558 | 0.043 |
| ***SVM-radial*** | 0.733 | 0.007 | 1 | 0 | 0.042 | 0.029 | 0.143 | 0.101 |
| ***RF*** | 0.881 | 0.02 | 0.964 | 0.001 | 0.667 | 0.064 | 0.691 | 0.05 |
| ***SVM-sigmoid*** | 0.718 | 0.017 | 0.875 | 0.024 | 0.313 | 0.026 | 0.222 | 0.034 |
| ***CART*** | 0.846 | 0.034 | 0.903 | 0.025 | 0.698 | 0.082 | 0.612 | 0.09 |
|  | ***Fitting (training set, n=81)*** | | | | | | | |
| ***Method*** | ***M Acc*** | ***SD Acc*** | ***M Sp*** | ***SD Sp*** | ***M Se*** | ***SD Se*** | ***M MCC*** | ***SD MCC*** |
| ***LDA*** | 0.99 | 0 | 1 | 0 | 0.961 | 0.003 | 0.973 | 0.001 |
| ***SVM-linear*** | 0.925 | 0.012 | 0.991 | 0.007 | 0.735 | 0.082 | 0.8 | 0.042 |
| ***NN*** | 0.973 | 0.017 | 0.991 | 0.006 | 0.925 | 0.048 | 0.931 | 0.042 |
| ***SVM-radial*** | 0.983 | 0.013 | 1 | 0 | 0.937 | 0.046 | 0.957 | 0.032 |
| ***RF*** | 0.99 | 0 | 1 | 0 | 0.961 | 0.003 | 0.973 | 0.001 |
| ***SVM-sigmoid*** | 0.686 | 0.026 | 0.81 | 0.013 | 0.331 | 0.139 | 0.142 | 0.129 |
| ***CART*** | 0.089 | 0.016 | 0.037 | 0.026 | 0.227 | 0.119 | -0.773 | 0.036 |
|  | ***External validation (test set, n=14)*** | | | | | | | |
| ***Method*** | ***M Acc*** | ***SD Acc*** | ***M Sp*** | ***SD Sp*** | ***M Se*** | ***SD Se*** | ***M MCC*** | ***SD MCC*** |
| ***LDA*** | 0.627 | 0.1 | 0.664 | 0.166 | 0.518 | 0.081 | 0.183 | 0.118 |
| ***SVM-linear*** | 0.804 | 0.028 | 0.974 | 0.036 | 0.524 | 0.034 | 0.574 | 0.079 |
| ***NN*** | 0.745 | 0.154 | 0.937 | 0.046 | 0.482 | 0.263 | 0.453 | 0.305 |
| ***SVM-radial*** | 0.627 | 0.1 | 1 | 0 | 0 | 0 | 0 | 0 |
| ***RF*** | 0.824 | 0.083 | 0.974 | 0.036 | 0.643 | 0.254 | 0.667 | 0.137 |
| ***SVM-sigmoid*** | 0.667 | 0.073 | 0.904 | 0.014 | 0.262 | 0.017 | 0.216 | 0.025 |
| ***CART*** | 0.275 | 0.1 | 0.17 | 0.116 | 0.512 | 0.19 | -0.343 | 0.206 |

**Table S5**: Performance of QSAR classification models for autofluorescence activity specific to wavelength and HepG2 cell-free assays. Each model building process was repeated 10 times with distinct data segregation and inactive undersampling from the entire Tox21 dataset, and the mean (M) and the standard deviation (SD) of each performance criterion are reported, Acc: accuracy, Sp: specificity, Se: sensitivity and MCC Matthew Coefficient Correlation, see methods.

| **Autofluorescence assays (HepG2 culture cell)** | | | | | | | | |
| --- | --- | --- | --- | --- | --- | --- | --- | --- |
| **Blue – cell free AC50** | | | | | | | | |
|  | ***10-fold cross-validation (full set, n=683)*** | | | | | | | |
| ***Method*** | ***M Acc*** | ***SD Acc*** | ***M Sp*** | ***SD Sp*** | ***M Se*** | ***SD Se*** | ***M MCC*** | ***SD MCC*** |
| ***LDA*** | 0.735 | 0.044 | 0.812 | 0.057 | 0.557 | 0.068 | 0.371 | 0.078 |
| ***SVM-linear*** | 0.798 | 0.036 | 0.912 | 0.042 | 0.541 | 0.082 | 0.499 | 0.089 |
| ***NN*** | 0.761 | 0.048 | 0.818 | 0.07 | 0.62 | 0.14 | 0.438 | 0.117 |
| ***SVM-radial*** | 0.71 | 0.04 | 0.999 | 0.004 | 0.043 | 0.026 | 0.143 | 0.083 |
| ***RF*** | 0.79 | 0.051 | 0.901 | 0.051 | 0.539 | 0.094 | 0.48 | 0.108 |
| ***SVM-sigmoid*** | 0.686 | 0.037 | 0.802 | 0.067 | 0.421 | 0.126 | 0.231 | 0.085 |
| ***CART*** | 0.249 | 0.04 | 0.174 | 0.059 | 0.421 | 0.074 | -0.41 | 0.086 |
|  | ***Fitting (training set, n=578)*** | | | | | | | |
| ***Method*** | ***M Acc*** | ***SD Acc*** | ***M Sp*** | ***SD Sp*** | ***M Se*** | ***SD Se*** | ***M MCC*** | ***SD MCC*** |
| ***LDA*** | 0.883 | 0.012 | 0.935 | 0.012 | 0.77 | 0.026 | 0.723 | 0.028 |
| ***SVM-linear*** | 0.829 | 0.007 | 0.93 | 0.019 | 0.607 | 0.047 | 0.586 | 0.018 |
| ***NN*** | 0.838 | 0.038 | 0.886 | 0.061 | 0.731 | 0.089 | 0.627 | 0.077 |
| ***SVM-radial*** | 0.975 | 0.005 | 1 | 0 | 0.92 | 0.016 | 0.942 | 0.011 |
| ***RF*** | 0.997 | 0.002 | 0.999 | 0.002 | 0.993 | 0.006 | 0.993 | 0.006 |
| ***SVM-sigmoid*** | 0.632 | 0.015 | 0.771 | 0.024 | 0.326 | 0.031 | 0.104 | 0.029 |
| ***CART*** | 0.123 | 0.009 | 0.065 | 0.018 | 0.248 | 0.047 | -0.71 | 0.024 |
|  | ***External validation (test set, n=105)*** | | | | | | | |
| ***Method*** | ***M Acc*** | ***SD Acc*** | ***M Sp*** | ***SD Sp*** | ***M Se*** | ***SD Se*** | ***M MCC*** | ***SD MCC*** |
| ***LDA*** | 0.735 | 0.044 | 0.812 | 0.057 | 0.557 | 0.068 | 0.371 | 0.078 |
| ***SVM-linear*** | 0.798 | 0.036 | 0.912 | 0.042 | 0.541 | 0.082 | 0.499 | 0.089 |
| ***NN*** | 0.761 | 0.048 | 0.818 | 0.07 | 0.62 | 0.14 | 0.438 | 0.117 |
| ***SVM-radial*** | 0.71 | 0.04 | 0.999 | 0.004 | 0.043 | 0.026 | 0.143 | 0.083 |
| ***RF*** | 0.79 | 0.051 | 0.901 | 0.051 | 0.539 | 0.094 | 0.48 | 0.108 |
| ***SVM-sigmoid*** | 0.686 | 0.037 | 0.802 | 0.067 | 0.421 | 0.126 | 0.231 | 0.085 |
| ***CART*** | 0.249 | 0.04 | 0.174 | 0.059 | 0.421 | 0.074 | -0.41 | 0.086 |
| **Green – cell free AC50** | | | | | | | | |
|  | ***10-fold cross-validation (full set, n=122)*** | | | | | | | |
| ***Method*** | ***M Acc*** | ***SD Acc*** | ***M Sp*** | ***SD Sp*** | ***M Se*** | ***SD Se*** | ***M MCC*** | ***SD MCC*** |
| ***LDA*** | 0.737 | 0.023 | 0.745 | 0.026 | 0.717 | 0.049 | 0.433 | 0.05 |
| ***SVM-linear*** | 0.807 | 0.021 | 0.941 | 0.017 | 0.484 | 0.045 | 0.5 | 0.061 |
| ***NN*** | 0.793 | 0.013 | 0.855 | 0.014 | 0.645 | 0.042 | 0.501 | 0.036 |
| ***SVM-radial*** | 0.722 | 0.007 | 1 | 0 | 0.054 | 0.01 | 0.197 | 0.02 |
| ***RF*** | 0.854 | 0.031 | 0.924 | 0.032 | 0.685 | 0.056 | 0.638 | 0.075 |
| ***SVM-sigmoid*** | 0.688 | 0.039 | 0.814 | 0.039 | 0.384 | 0.064 | 0.211 | 0.097 |
| ***CART*** | 0.805 | 0.022 | 0.869 | 0.028 | 0.65 | 0.044 | 0.527 | 0.049 |
|  | ***Fitting (training set, n=104)*** | | | | | | | |
| ***Method*** | ***M Acc*** | ***SD Acc*** | ***M Sp*** | ***SD Sp*** | ***M Se*** | ***SD Se*** | ***M MCC*** | ***SD MCC*** |
| ***LDA*** | 0.998 | 0.004 | 0.998 | 0.003 | 0.996 | 0.007 | 0.994 | 0.008 |
| ***SVM-linear*** | 0.891 | 0.017 | 0.97 | 0.013 | 0.701 | 0.04 | 0.731 | 0.044 |
| ***NN*** | 0.936 | 0.023 | 0.95 | 0.029 | 0.901 | 0.064 | 0.849 | 0.053 |
| ***SVM-radial*** | 0.982 | 0.005 | 1 | 0 | 0.941 | 0.016 | 0.958 | 0.011 |
| ***RF*** | 0.994 | 0.007 | 0.996 | 0.007 | 0.989 | 0.012 | 0.987 | 0.017 |
| ***SVM-sigmoid*** | 0.649 | 0.042 | 0.782 | 0.042 | 0.331 | 0.083 | 0.119 | 0.114 |
| ***CART*** | 0.11 | 0.013 | 0.065 | 0.034 | 0.221 | 0.073 | -0.735 | 0.031 |
|  | ***External validation (test set, n=18)*** | | | | | | | |
| ***Method*** | ***M Acc*** | ***SD Acc*** | ***M Sp*** | ***SD Sp*** | ***M Se*** | ***SD Se*** | ***M MCC*** | ***SD MCC*** |
| ***LDA*** | 0.695 | 0.066 | 0.708 | 0.07 | 0.664 | 0.156 | 0.335 | 0.15 |
| ***SVM-linear*** | 0.808 | 0.087 | 0.918 | 0.053 | 0.558 | 0.191 | 0.51 | 0.166 |
| ***NN*** | 0.805 | 0.063 | 0.845 | 0.062 | 0.699 | 0.081 | 0.528 | 0.121 |
| ***SVM-radial*** | 0.737 | 0.081 | 1 | 0 | 0.056 | 0.058 | 0.146 | 0.148 |
| ***RF*** | 0.851 | 0.075 | 0.912 | 0.091 | 0.709 | 0.124 | 0.643 | 0.138 |
| ***SVM-sigmoid*** | 0.688 | 0.065 | 0.827 | 0.07 | 0.317 | 0.089 | 0.163 | 0.122 |
| ***CART*** | 0.184 | 0.06 | 0.129 | 0.079 | 0.325 | 0.136 | -0.548 | 0.101 |
| **Red – cell free AC50** | | | | | | | | |
|  | ***10-fold cross-validation (full set, n=101)*** | | | | | | | |
| ***Method*** | ***M Acc*** | ***SD Acc*** | ***M Sp*** | ***SD Sp*** | ***M Se*** | ***SD Se*** | ***M MCC*** | ***SD MCC*** |
| ***LDA*** | 0.753 | 0.03 | 0.761 | 0.023 | 0.734 | 0.073 | 0.458 | 0.072 |
| ***SVM-linear*** | 0.859 | 0.012 | 0.956 | 0.005 | 0.613 | 0.04 | 0.635 | 0.026 |
| ***NN*** | 0.803 | 0.025 | 0.854 | 0.053 | 0.669 | 0.083 | 0.522 | 0.053 |
| ***SVM-radial*** | 0.755 | 0.007 | 1 | 0 | 0.129 | 0 | 0.31 | 0.002 |
| ***RF*** | 0.873 | 0.021 | 0.949 | 0.024 | 0.677 | 0.023 | 0.675 | 0.053 |
| ***SVM-sigmoid*** | 0.725 | 0.058 | 0.828 | 0.037 | 0.46 | 0.122 | 0.296 | 0.161 |
| ***CART*** | 0.787 | 0.017 | 0.864 | 0.028 | 0.589 | 0.027 | 0.464 | 0.031 |
|  | ***Fitting (training set, n=86)*** | | | | | | | |
| ***Method*** | ***M Acc*** | ***SD Acc*** | ***M Sp*** | ***SD Sp*** | ***M Se*** | ***SD Se*** | ***M MCC*** | ***SD MCC*** |
| ***LDA*** | 0.992 | 0.009 | 0.996 | 0.006 | 0.981 | 0.019 | 0.98 | 0.021 |
| ***SVM-linear*** | 0.955 | 0.031 | 0.981 | 0.012 | 0.894 | 0.075 | 0.891 | 0.075 |
| ***NN*** | 0.974 | 0.04 | 0.996 | 0.006 | 0.925 | 0.13 | 0.94 | 0.09 |
| ***SVM-radial*** | 0.981 | 0.01 | 1 | 0 | 0.936 | 0.029 | 0.955 | 0.021 |
| ***RF*** | 0.994 | 0.01 | 0.996 | 0.007 | 0.991 | 0.016 | 0.987 | 0.023 |
| ***SVM-sigmoid*** | 0.683 | 0.07 | 0.787 | 0.047 | 0.422 | 0.135 | 0.212 | 0.182 |
| ***CART*** | 0.091 | 0.011 | 0.065 | 0.036 | 0.16 | 0.068 | -0.782 | 0.015 |
|  | ***External validation (test set, n=15)*** | | | | | | | |
| ***Method*** | ***M Acc*** | ***SD Acc*** | ***M Sp*** | ***SD Sp*** | ***M Se*** | ***SD Se*** | ***M MCC*** | ***SD MCC*** |
| ***LDA*** | 0.733 | 0.133 | 0.807 | 0.113 | 0.634 | 0.268 | 0.365 | 0.25 |
| ***SVM-linear*** | 0.895 | 0.087 | 0.954 | 0.046 | 0.768 | 0.153 | 0.753 | 0.183 |
| ***NN*** | 0.835 | 0.088 | 0.873 | 0.058 | 0.58 | 0.368 | 0.431 | 0.333 |
| ***SVM-radial*** | 0.791 | 0.147 | 1 | 0 | 0.313 | 0.41 | 0.362 | 0.411 |
| ***RF*** | 0.865 | 0.089 | 0.979 | 0.036 | 0.607 | 0.229 | 0.668 | 0.204 |
| ***SVM-sigmoid*** | 0.791 | 0.027 | 0.888 | 0.044 | 0.393 | 0.229 | 0.332 | 0.259 |
| ***CART*** | 0.208 | 0.12 | 0.118 | 0.082 | 0.366 | 0.268 | -0.469 | 0.23 |

**Table S6**: Performance of QSAR classification models for autofluorescence activity specific to wavelength and Hek293 cell-based assays. Each model building process was repeated 10 times with distinct data segregation and inactive undersampling from the entire Tox21 dataset, and the mean (M) and the standard deviation (SD) of each performance criterion are reported, Acc: accuracy, Sp: specificity, Se: sensitivity and MCC Matthew Coefficient Correlation, see methods.

| **Autofluorescence assays (Hek293 culture cell)** | | | | | | | | |
| --- | --- | --- | --- | --- | --- | --- | --- | --- |
| **Blue – cell based AC50** | | | | | | | | |
|  | ***10-fold cross-validation (full set, n=670)*** | | | | | | | |
| ***Method*** | ***M Acc*** | ***SD Acc*** | ***M Sp*** | ***SD Sp*** | ***M Se*** | ***SD Se*** | ***M MCC*** | ***SD MCC*** |
| ***LDA*** | 0.771 | 0.019 | 0.844 | 0.016 | 0.61 | 0.031 | 0.46 | 0.043 |
| ***SVM-linear*** | 0.79 | 0.015 | 0.91 | 0.016 | 0.524 | 0.016 | 0.482 | 0.036 |
| ***NN*** | 0.757 | 0.023 | 0.805 | 0.021 | 0.649 | 0.055 | 0.446 | 0.057 |
| ***SVM-radial*** | 0.708 | 0.004 | 0.998 | 0.002 | 0.063 | 0.008 | 0.197 | 0.021 |
| ***RF*** | 0.793 | 0.011 | 0.91 | 0.012 | 0.533 | 0.037 | 0.489 | 0.029 |
| ***SVM-sigmoid*** | 0.674 | 0.025 | 0.817 | 0.027 | 0.355 | 0.038 | 0.188 | 0.059 |
| ***CART*** | 0.742 | 0.015 | 0.832 | 0.019 | 0.543 | 0.028 | 0.384 | 0.033 |
|  | ***Fitting (training set, n=570)*** | | | | | | | |
| ***Method*** | ***M Acc*** | ***SD Acc*** | ***M Sp*** | ***SD Sp*** | ***M Se*** | ***SD Se*** | ***M MCC*** | ***SD MCC*** |
| ***LDA*** | 0.894 | 0.013 | 0.943 | 0.014 | 0.784 | 0.021 | 0.748 | 0.027 |
| ***SVM-linear*** | 0.835 | 0.013 | 0.936 | 0.014 | 0.608 | 0.057 | 0.597 | 0.037 |
| ***NN*** | 0.846 | 0.033 | 0.881 | 0.044 | 0.766 | 0.078 | 0.645 | 0.074 |
| ***SVM-radial*** | 0.973 | 0.006 | 1 | 0 | 0.913 | 0.018 | 0.937 | 0.013 |
| ***RF*** | 0.998 | 0.001 | 1 | 0.001 | 0.995 | 0.004 | 0.996 | 0.003 |
| ***SVM-sigmoid*** | 0.636 | 0.017 | 0.773 | 0.025 | 0.33 | 0.023 | 0.109 | 0.024 |
| ***CART*** | 0.113 | 0.006 | 0.064 | 0.016 | 0.224 | 0.041 | -0.731 | 0.013 |
|  | ***External validation (test set, n=100)*** | | | | | | | |
| ***Method*** | ***M Acc*** | ***SD Acc*** | ***M Sp*** | ***SD Sp*** | ***M Se*** | ***SD Se*** | ***M MCC*** | ***SD MCC*** |
| ***LDA*** | 0.766 | 0.032 | 0.824 | 0.042 | 0.635 | 0.078 | 0.457 | 0.077 |
| ***SVM-linear*** | 0.792 | 0.026 | 0.906 | 0.028 | 0.537 | 0.057 | 0.487 | 0.068 |
| ***NN*** | 0.732 | 0.054 | 0.797 | 0.056 | 0.588 | 0.128 | 0.383 | 0.11 |
| ***SVM-radial*** | 0.699 | 0.049 | 0.998 | 0.005 | 0.054 | 0.032 | 0.165 | 0.093 |
| ***RF*** | 0.789 | 0.032 | 0.9 | 0.029 | 0.541 | 0.089 | 0.48 | 0.091 |
| ***SVM-sigmoid*** | 0.658 | 0.045 | 0.802 | 0.021 | 0.348 | 0.133 | 0.154 | 0.129 |
| ***CART*** | 0.257 | 0.034 | 0.173 | 0.047 | 0.445 | 0.098 | -0.391 | 0.095 |
| ***Green – cell based AC50*** | | | | | | | | |
|  | ***10-fold cross-validation (full set, n=238)*** | | | | | | | |
| ***Method*** | ***M Acc*** | ***SD Acc*** | ***M Sp*** | ***SD Sp*** | ***M Se*** | ***SD Se*** | ***M MCC*** | ***SD MCC*** |
| ***LDA*** | 0.618 | 0.036 | 0.614 | 0.041 | 0.628 | 0.072 | 0.22 | 0.075 |
| ***SVM-linear*** | 0.835 | 0.011 | 0.956 | 0.013 | 0.534 | 0.053 | 0.573 | 0.034 |
| ***NN*** | 0.802 | 0.036 | 0.859 | 0.042 | 0.657 | 0.103 | 0.518 | 0.089 |
| ***SVM-radial*** | 0.737 | 0.009 | 1 | 0 | 0.081 | 0.013 | 0.242 | 0.023 |
| ***RF*** | 0.852 | 0.022 | 0.956 | 0.017 | 0.594 | 0.069 | 0.621 | 0.056 |
| ***SVM-sigmoid*** | 0.688 | 0.016 | 0.847 | 0.028 | 0.291 | 0.06 | 0.159 | 0.055 |
| ***CART*** | 0.766 | 0.033 | 0.851 | 0.039 | 0.555 | 0.052 | 0.419 | 0.07 |
|  | ***Fitting (training set, n=202)*** | | | | | | | |
| ***Method*** | ***M Acc*** | ***SD Acc*** | ***M Sp*** | ***SD Sp*** | ***M Se*** | ***SD Se*** | ***M MCC*** | ***SD MCC*** |
| ***LDA*** | 1 | 0 | 1 | 0 | 1 | 0 | 1 | 0 |
| ***SVM-linear*** | 0.912 | 0.047 | 0.978 | 0.015 | 0.75 | 0.139 | 0.78 | 0.117 |
| ***NN*** | 0.961 | 0.02 | 0.974 | 0.02 | 0.927 | 0.068 | 0.905 | 0.05 |
| ***SVM-radial*** | 0.978 | 0.007 | 1 | 0 | 0.924 | 0.026 | 0.947 | 0.018 |
| ***RF*** | 0.999 | 0.003 | 1 | 0 | 0.995 | 0.01 | 0.997 | 0.007 |
| ***SVM-sigmoid*** | 0.652 | 0.016 | 0.789 | 0.024 | 0.311 | 0.059 | 0.106 | 0.063 |
| ***CART*** | 0.105 | 0.017 | 0.074 | 0.029 | 0.182 | 0.058 | -0.745 | 0.043 |
|  | ***External validation (test set, n=36)*** | | | | | | | |
| ***Method*** | ***M Acc*** | ***SD Acc*** | ***M Sp*** | ***SD Sp*** | ***M Se*** | ***SD Se*** | ***M MCC*** | ***SD MCC*** |
| ***LDA*** | 0.611 | 0.095 | 0.595 | 0.132 | 0.628 | 0.179 | 0.205 | 0.153 |
| ***SVM-linear*** | 0.849 | 0.05 | 0.943 | 0.034 | 0.614 | 0.134 | 0.597 | 0.106 |
| ***NN*** | 0.739 | 0.113 | 0.829 | 0.129 | 0.583 | 0.159 | 0.409 | 0.193 |
| ***SVM-radial*** | 0.743 | 0.119 | 1 | 0 | 0.132 | 0.119 | 0.259 | 0.201 |
| ***RF*** | 0.882 | 0.03 | 0.963 | 0.042 | 0.679 | 0.077 | 0.686 | 0.083 |
| ***SVM-sigmoid*** | 0.675 | 0.103 | 0.809 | 0.103 | 0.275 | 0.242 | 0.086 | 0.229 |
| ***CART*** | 0.203 | 0.065 | 0.147 | 0.08 | 0.317 | 0.157 | -0.515 | 0.151 |
| ***Red – cell based AC50*** | | | | | | | | |
|  | ***10-fold cross-validation (full set, n=116)*** | | | | | | | |
| ***Method*** | ***M Acc*** | ***SD Acc*** | ***M Sp*** | ***SD Sp*** | ***M Se*** | ***SD Se*** | ***M MCC*** | ***SD MCC*** |
| ***LDA*** | 0.797 | 0.034 | 0.802 | 0.041 | 0.786 | 0.044 | 0.553 | 0.067 |
| ***SVM-linear*** | 0.835 | 0.032 | 0.92 | 0.051 | 0.625 | 0.018 | 0.589 | 0.069 |
| ***NN*** | 0.828 | 0.049 | 0.899 | 0.029 | 0.652 | 0.102 | 0.568 | 0.13 |
| ***SVM-radial*** | 0.727 | 0.01 | 1 | 0 | 0.054 | 0.031 | 0.171 | 0.099 |
| ***RF*** | 0.877 | 0.034 | 0.936 | 0.024 | 0.732 | 0.096 | 0.692 | 0.09 |
| ***SVM-sigmoid*** | 0.712 | 0.058 | 0.808 | 0.037 | 0.473 | 0.125 | 0.284 | 0.15 |
| ***CART*** | 0.797 | 0.048 | 0.848 | 0.018 | 0.67 | 0.125 | 0.509 | 0.129 |
|  | ***Fitting (training set, n=99)*** | | | | | | | |
| ***Method*** | ***M Acc*** | ***SD Acc*** | ***M Sp*** | ***SD Sp*** | ***M Se*** | ***SD Se*** | ***M MCC*** | ***SD MCC*** |
| ***LDA*** | 1 | 0 | 1 | 0 | 1 | 0 | 1 | 0 |
| ***SVM-linear*** | 0.949 | 0.028 | 0.983 | 0.013 | 0.868 | 0.093 | 0.876 | 0.071 |
| ***NN*** | 0.964 | 0.037 | 1 | 0 | 0.871 | 0.131 | 0.91 | 0.091 |
| ***SVM-radial*** | 0.979 | 0.005 | 1 | 0 | 0.929 | 0.017 | 0.949 | 0.012 |
| ***RF*** | 0.994 | 0.006 | 1 | 0 | 0.98 | 0.02 | 0.986 | 0.014 |
| ***SVM-sigmoid*** | 0.69 | 0.108 | 0.787 | 0.076 | 0.468 | 0.232 | 0.252 | 0.252 |
| ***CART*** | 0.085 | 0.01 | 0.069 | 0.04 | 0.121 | 0.07 | -0.805 | 0.008 |
|  | ***External validation (test set, n=17)*** | | | | | | | |
| ***Method*** | ***M Acc*** | ***SD Acc*** | ***M Sp*** | ***SD Sp*** | ***M Se*** | ***SD Se*** | ***M MCC*** | ***SD MCC*** |
| ***LDA*** | 0.846 | 0.071 | 0.841 | 0.034 | 0.9 | 0.173 | 0.624 | 0.173 |
| ***SVM-linear*** | 0.882 | 0.128 | 0.954 | 0.048 | 0.75 | 0.433 | 0.636 | 0.384 |
| ***NN*** | 0.794 | 0.08 | 0.887 | 0.082 | 0.417 | 0.26 | 0.321 | 0.285 |
| ***SVM-radial*** | 0.757 | 0.115 | 1 | 0 | 0 | 0 | 0 | 0 |
| ***RF*** | 0.848 | 0.107 | 0.887 | 0.073 | 0.767 | 0.252 | 0.598 | 0.252 |
| ***SVM-sigmoid*** | 0.724 | 0.07 | 0.853 | 0.105 | 0.483 | 0.307 | 0.296 | 0.205 |
| ***CART*** | 0.17 | 0.072 | 0.076 | 0.088 | 0.333 | 0.294 | -0.561 | 0.18 |

**Table S7**: Performance of QSAR classification models for autofluorescence activity specific to wavelength and Hek293 cell-free assays. Each model building process was repeated 10 times with distinct data segregation and inactive undersampling from the entire Tox21 dataset, and the mean (M) and the standard deviation (SD) of each performance criterion are reported, Acc: accuracy, Sp: specificity, Se: sensitivity and MCC Matthew Coefficient Correlation, see methods.

| **Autofluorescence assays (Hek293 culture cell)** | | | | | | | | |
| --- | --- | --- | --- | --- | --- | --- | --- | --- |
| **Blue – cell free AC50** | | | | | | | | |
|  | ***10-fold cross-validation (full set, n=633)*** | | | | | | | |
| ***Method*** | ***M Acc*** | ***SD Acc*** | ***M Sp*** | ***SD Sp*** | ***M Se*** | ***SD Se*** | ***M MCC*** | ***SD MCC*** |
| ***LDA*** | 0.751 | 0.009 | 0.838 | 0.007 | 0.555 | 0.025 | 0.403 | 0.023 |
| ***SVM-linear*** | 0.778 | 0.01 | 0.911 | 0.01 | 0.482 | 0.022 | 0.445 | 0.028 |
| ***NN*** | 0.723 | 0.018 | 0.807 | 0.021 | 0.535 | 0.053 | 0.346 | 0.048 |
| ***SVM-radial*** | 0.708 | 0.004 | 0.997 | 0.002 | 0.061 | 0.007 | 0.19 | 0.014 |
| ***RF*** | 0.784 | 0.016 | 0.908 | 0.017 | 0.505 | 0.032 | 0.462 | 0.04 |
| ***SVM-sigmoid*** | 0.657 | 0.021 | 0.793 | 0.021 | 0.354 | 0.025 | 0.156 | 0.047 |
| ***CART*** | 0.738 | 0.013 | 0.841 | 0.014 | 0.507 | 0.024 | 0.364 | 0.03 |
|  | ***Fitting (training set, n=538)*** | | | | | | | |
| ***Method*** | ***M Acc*** | ***SD Acc*** | ***M Sp*** | ***SD Sp*** | ***M Se*** | ***SD Se*** | ***M MCC*** | ***SD MCC*** |
| ***LDA*** | 0.873 | 0.011 | 0.931 | 0.009 | 0.741 | 0.028 | 0.694 | 0.026 |
| ***SVM-linear*** | 0.833 | 0.008 | 0.944 | 0.009 | 0.583 | 0.028 | 0.589 | 0.02 |
| ***NN*** | 0.792 | 0.048 | 0.827 | 0.08 | 0.712 | 0.062 | 0.535 | 0.081 |
| ***SVM-radial*** | 0.974 | 0.007 | 1 | 0 | 0.917 | 0.023 | 0.94 | 0.017 |
| ***RF*** | 0.998 | 0.002 | 0.999 | 0.002 | 0.995 | 0.005 | 0.995 | 0.004 |
| ***SVM-sigmoid*** | 0.616 | 0.033 | 0.755 | 0.038 | 0.302 | 0.038 | 0.06 | 0.063 |
| ***CART*** | 0.131 | 0.012 | 0.064 | 0.009 | 0.28 | 0.045 | -0.685 | 0.033 |
|  | ***External validation (test set, n=95)*** | | | | | | | |
| ***Method*** | ***M Acc*** | ***SD Acc*** | ***M Sp*** | ***SD Sp*** | ***M Se*** | ***SD Se*** | ***M MCC*** | ***SD MCC*** |
| ***LDA*** | 0.745 | 0.038 | 0.832 | 0.06 | 0.559 | 0.134 | 0.402 | 0.095 |
| ***SVM-linear*** | 0.783 | 0.039 | 0.925 | 0.028 | 0.481 | 0.142 | 0.466 | 0.11 |
| ***NN*** | 0.709 | 0.033 | 0.775 | 0.089 | 0.572 | 0.122 | 0.348 | 0.06 |
| ***SVM-radial*** | 0.705 | 0.043 | 0.999 | 0.004 | 0.076 | 0.044 | 0.212 | 0.084 |
| ***RF*** | 0.782 | 0.038 | 0.913 | 0.045 | 0.507 | 0.093 | 0.475 | 0.079 |
| ***SVM-sigmoid*** | 0.662 | 0.041 | 0.803 | 0.063 | 0.369 | 0.111 | 0.186 | 0.101 |
| ***CART*** | 0.263 | 0.039 | 0.148 | 0.055 | 0.512 | 0.105 | -0.363 | 0.093 |
| ***Green – cell free AC50*** | | | | | | | | |
|  | ***10-fold cross-validation (full set, n=244)*** | | | | | | | |
| ***Method*** | ***M Acc*** | ***SD Acc*** | ***M Sp*** | ***SD Sp*** | ***M Se*** | ***SD Se*** | ***M MCC*** | ***SD MCC*** |
| ***LDA*** | 0.701 | 0.054 | 0.721 | 0.066 | 0.651 | 0.063 | 0.345 | 0.093 |
| ***SVM-linear*** | 0.839 | 0.028 | 0.927 | 0.03 | 0.61 | 0.063 | 0.582 | 0.071 |
| ***NN*** | 0.776 | 0.036 | 0.849 | 0.038 | 0.584 | 0.065 | 0.439 | 0.079 |
| ***SVM-radial*** | 0.733 | 0.009 | 1 | 0 | 0.038 | 0.027 | 0.136 | 0.097 |
| ***RF*** | 0.848 | 0.028 | 0.953 | 0.019 | 0.575 | 0.077 | 0.599 | 0.072 |
| ***SVM-sigmoid*** | 0.711 | 0.029 | 0.841 | 0.03 | 0.371 | 0.06 | 0.231 | 0.072 |
| ***CART*** | 0.783 | 0.029 | 0.883 | 0.026 | 0.524 | 0.085 | 0.433 | 0.079 |
|  | ***Fitting (training set, n=207)*** | | | | | | | |
| ***Method*** | ***M Acc*** | ***SD Acc*** | ***M Sp*** | ***SD Sp*** | ***M Se*** | ***SD Se*** | ***M MCC*** | ***SD MCC*** |
| ***LDA*** | 0.998 | 0.004 | 1 | 0 | 0.993 | 0.014 | 0.995 | 0.01 |
| ***SVM-linear*** | 0.955 | 0.035 | 0.991 | 0.011 | 0.861 | 0.108 | 0.887 | 0.087 |
| ***NN*** | 0.953 | 0.03 | 0.984 | 0.013 | 0.875 | 0.09 | 0.883 | 0.074 |
| ***SVM-radial*** | 0.984 | 0.009 | 1 | 0 | 0.944 | 0.031 | 0.961 | 0.022 |
| ***RF*** | 0.996 | 0.008 | 1 | 0 | 0.986 | 0.027 | 0.99 | 0.019 |
| ***SVM-sigmoid*** | 0.698 | 0.032 | 0.802 | 0.027 | 0.427 | 0.079 | 0.233 | 0.083 |
| ***CART*** | 0.104 | 0.016 | 0.038 | 0.037 | 0.277 | 0.093 | -0.739 | 0.038 |
|  | ***External validation (test set, n=37)*** | | | | | | | |
| ***Method*** | ***M Acc*** | ***SD Acc*** | ***M Sp*** | ***SD Sp*** | ***M Se*** | ***SD Se*** | ***M MCC*** | ***SD MCC*** |
| ***LDA*** | 0.683 | 0.084 | 0.721 | 0.098 | 0.601 | 0.124 | 0.292 | 0.142 |
| ***SVM-linear*** | 0.859 | 0.067 | 0.919 | 0.092 | 0.652 | 0.21 | 0.634 | 0.144 |
| ***NN*** | 0.825 | 0.089 | 0.888 | 0.101 | 0.631 | 0.148 | 0.553 | 0.215 |
| ***SVM-radial*** | 0.736 | 0.088 | 1 | 0 | 0 | 0 | 0 | 0 |
| ***RF*** | 0.847 | 0.042 | 0.968 | 0.047 | 0.476 | 0.145 | 0.56 | 0.141 |
| ***SVM-sigmoid*** | 0.731 | 0.069 | 0.842 | 0.061 | 0.434 | 0.156 | 0.279 | 0.152 |
| ***CART*** | 0.183 | 0.071 | 0.091 | 0.071 | 0.435 | 0.205 | -0.515 | 0.143 |
| ***Red – cell free AC50*** | | | | | | | | |
|  | ***10-fold cross-validation (full set, n=109)*** | | | | | | | |
| ***Method*** | ***M Acc*** | ***SD Acc*** | ***M Sp*** | ***SD Sp*** | ***M Se*** | ***SD Se*** | ***M MCC*** | ***SD MCC*** |
| ***LDA*** | 0.727 | 0.04 | 0.756 | 0.028 | 0.655 | 0.069 | 0.387 | 0.089 |
| ***SVM-linear*** | 0.856 | 0.002 | 0.965 | 0.006 | 0.586 | 0 | 0.632 | 0.012 |
| ***NN*** | 0.826 | 0.018 | 0.875 | 0.01 | 0.707 | 0.086 | 0.579 | 0.055 |
| ***SVM-radial*** | 0.723 | 0.004 | 1 | 0 | 0.034 | 0.034 | 0.112 | 0.112 |
| ***RF*** | 0.891 | 0.002 | 0.972 | 0.001 | 0.69 | 0 | 0.725 | 0.002 |
| ***SVM-sigmoid*** | 0.754 | 0.055 | 0.869 | 0.017 | 0.466 | 0.155 | 0.354 | 0.17 |
| ***CART*** | 0.807 | 0.019 | 0.875 | 0.017 | 0.638 | 0.017 | 0.521 | 0.038 |
|  | ***Fitting (training set, n=93)*** | | | | | | | |
| ***Method*** | ***M Acc*** | ***SD Acc*** | ***M Sp*** | ***SD Sp*** | ***M Se*** | ***SD Se*** | ***M MCC*** | ***SD MCC*** |
| ***LDA*** | 1 | 0 | 1 | 0 | 1 | 0 | 1 | 0 |
| ***SVM-linear*** | 0.901 | 0.008 | 0.966 | 0 | 0.753 | 0.033 | 0.762 | 0.025 |
| ***NN*** | 0.977 | 0.023 | 0.983 | 0.017 | 0.964 | 0.036 | 0.948 | 0.052 |
| ***SVM-radial*** | 0.977 | 0.011 | 1 | 0 | 0.926 | 0.034 | 0.947 | 0.025 |
| ***RF*** | 1 | 0 | 1 | 0 | 1 | 0 | 1 | 0 |
| ***SVM-sigmoid*** | 0.786 | 0.047 | 0.824 | 0.024 | 0.704 | 0.096 | 0.513 | 0.11 |
| ***CART*** | 0.098 | 0.015 | 0.05 | 0 | 0.205 | 0.045 | -0.765 | 0.033 |
|  | ***External validation (test set, n=16)*** | | | | | | | |
| ***Method*** | ***M Acc*** | ***SD Acc*** | ***M Sp*** | ***SD Sp*** | ***M Se*** | ***SD Se*** | ***M MCC*** | ***SD MCC*** |
| ***LDA*** | 0.767 | 0.033 | 0.883 | 0.026 | 0.125 | 0.125 | 0.051 | 0.156 |
| ***SVM-linear*** | 0.833 | 0.1 | 0.955 | 0.045 | 0.125 | 0.125 | 0.103 | 0.103 |
| ***NN*** | 0.733 | 0 | 0.756 | 0.029 | 0.375 | 0.375 | 0.149 | 0.282 |
| ***SVM-radial*** | 0.833 | 0.1 | 1 | 0 | 0 | 0 | 0 | 0 |
| ***RF*** | 0.933 | 0 | 0.955 | 0.045 | 0.5 | 0.5 | 0.426 | 0.426 |
| ***SVM-sigmoid*** | 0.833 | 0.033 | 0.873 | 0.055 | 0.375 | 0.375 | 0.231 | 0.302 |
| ***CART*** | 0.2 | 0.133 | 0.091 | 0.091 | 0.875 | 0.125 | -0.038 | 0.038 |

**Table S8**: Summary of the top 10 molecular descriptors included in the QSAR models developed from autofluorescence assays for each color channel, all color channels combined, and for the luciferase inhibition assay.

| **Descriptor name** | **Description** | **Reference** | **QSAR models** |
| --- | --- | --- | --- |
| **Physicochemical descriptors** | | | |
| UI | Unsaturation index | RDKit | - Luciferase QSAR  - All colors QSAR  - Blue QSAR  - Red QSAR |
| logP_pred | Octanol–water partition coefficient prediction | OPERA | - Luciferase QSAR |
| BP_pred | Boiling point prediction | OPERA | - Luciferase QSAR  - Green QSAR  - Red QSAR |
| MP_pred | Melting point prediction | OPERA | - Blue QSAR  - Green QSAR  - Red QSAR |
| BioDeg_LogHalfLife_pred | Biodegradability half-life | OPERA | - Red QSAR |
| **Constitutional descriptors** | | | |
| Sp3Sp2HybRatio | Sp3 and Sp2 ratio bonds | OPERA | - Luciferase QSAR |
| CombDipolPolariz | Combined dipolarity/polarizability | OPERA | - All colors QSAR  - Blue QSAR  - Green QSAR |
| naro | number of aromatic bonds | RDKit | - Luciferase QSAR |
| **Charge descriptors** | | | |
| QNmin | Most negative charge on N atoms | RDKit | - All colors QSAR  - Green QSAR  - Red QSAR |
| Qmin | Most negative charge in a molecule | RDKit | - Red QSAR |
| QOmin | Most negative charge on O atoms | RDKit | - Red QSAR |
| **Basak descriptor** | | | |
| IC1 | “Steric hindrance” around a path of two heteroatoms | RDKit | - Blue QSAR |
| **Topological descriptors** | | | |
| BertzCT | Bertz complexity index | RDKit | - All colors QSAR  - Blue QSAR  - Green QSAR  - Red QSAR |
| J | Balaban’s J index | RDKit | - Luciferase QSAR |
| **Connectivity descriptors** | | | |
| dchi3 | Difference between the simple molecular connectivity Chi indices and the valence of molecular connectivity for cycles of 3 | RDKit | - All colors QSAR  - Blue QSAR  - Green QSAR |
| Chi6ch | Simple molecular connectivity Chi indices for cycles of 6 | RDKit | - All colors QSAR |
| **E-State descriptors** | | | |
| S12 | Sum of E-State of atom type: aaCH | RDKit | - Luciferase QSAR |
| S18 | Sum of E-State of atom type: aaaC | RDKit | - All colors QSAR  - Blue QSAR |
| S21 | Sum of E-State of atom type: sNH2 | RDKit | - Red QSAR |
| **MOE-type descriptors** | | | |
| POEOEVSA0 | MOE-type descriptors using partial charges and surface area contributions | RDKit | - Green QSAR |
| MRVSA9 | MOE-type descriptors using molecular refractivity contributions and surface area contributions | RDKit | - Luciferase QSAR |
| slogPVSA7 | MOE-type descriptors using SLogP contributions and surface area contributions | RDKit | - All colors QSAR  - Blue QSAR |
| EstateVSA9 | MOE-type descriptors using Estate indices and surface area contributions | RDKit | - Red QSAR |
| **Burden descriptors** | | | |
| bcutp1 | Lowest eigen value 1 of Burden matrix/weighted by atomic polarizabilities | RDKit | - All colors QSAR  - Blue QSAR  - Green QSAR  - Red QSAR |
| bcutp2 | Lowest eigen value 2 of Burden matrix/weighted by atomic polarizabilities | RDKit | - Luciferase QSAR |
| bcutp9 | Lowest eigen value 9 of Burden matrix/weighted by atomic polarizabilities | RDKit | - All colors QSAR  - Blue QSAR  - Green QSAR |
| bcutp10 | Lowest eigen value 10 of Burden matrix/weighted by atomic polarizabilities |  | - Luciferase QSAR |
| **Fingerprints** | | | |
| Sfinger17 | Daylight-like topological fingerprint based on hashing molecular subgraphs | RDKit | - Green QSAR  - Red QSAR |

**Table S9**: Mean for active and inactive chemicals for the top10 descriptors involved in the RF models, see Table S8. Student test significativity between active and inactive chemicals is presented by (***) for p-value < 0.001, (**) for p-values < 0.01 and (*) for p-values < 0.05.

| **Descriptor** | **Luciferase** | | | **Cross colors** | | | **Blue** | | | **Green** | | | **Red** | | |
| --- | --- | --- | --- | --- | --- | --- | --- | --- | --- | --- | --- | --- | --- | --- | --- |
|  | **Mact** | **Minac** | **pval** | **Mact** | **Minac** | **pval** | **Mact** | **Minac** | **pval** | **Mact** | **Minac** | **pval** | **Mact** | **Minac** | **pval** |
| UI | 3.56 | 2.62 | *** | 3.77 | 2.65 | ******* | 3.74 | 2.66 | ******* | 3.9 | 2.67 | ******* | 4.17 | 2.67 | ******* |
| logP_pred | 3.14 | 2.33 | *** | 2.33 | 2.37 | **-** | 2.25 | 2.38 | **-** | 2.51 | 2.37 | **-** | 2.96 | 2.37 | **-** |
| BP_pred | 330.19 | 289.94 | *** | 353.61 | 291.13 | ******* | 348.06 | 291.42 | ******* | 378.33 | 291.98 | ******* | 402.63 | 292.03 | ******* |
| MP_pred | 135.84 | 95.44 | *** | 175.07 | 96.34 | ******* | 170.82 | 96.66 | ******* | 190.27 | 97.47 | ******* | 219.31 | 97.51 | ******* |
| BioDeg_LogHalfLife_pred | 0.87 | 0.79 | *** | 1.03 | 0.79 | ******* | 0.95 | 0.79 | ******* | 1.31 | 0.79 | ******* | 1.53 | 0.79 | ******* |
| Sp3Sp2HybRatio | 0.21 | 0.48 | *** | 0.28 | 0.47 | ******* | 0.28 | 0.47 | ******* | 0.3 | 0.47 | ****** | 0.24 | 0.47 | ******* |
| CombDipolPolariz | 2.01 | 1.69 | *** | 2.83 | 1.69 | ******* | 2.81 | 1.69 | ******* | 3.1 | 1.7 | ******* | 3.24 | 1.71 | ******* |
| naro | 11.12 | 6.06 | *** | 12.05 | 6.25 | ******* | 11.76 | 6.27 | ******* | 13.5 | 6.33 | ******* | 15.15 | 6.34 | ******* |
| QNmin | -0.19 | -0.18 | - | -0.26 | -0.18 | ******* | -0.27 | -0.18 | ******* | -0.26 | -0.18 | ***** | -0.22 | -0.18 | **-** |
| Qmin | -0.43 | -0.4 | *** | -0.46 | -0.4 | ******* | -0.45 | -0.4 | ******* | -0.52 | -0.4 | ******* | -0.57 | -0.4 | ******* |
| QOmin | -0.36 | -0.35 | - | -0.4 | -0.35 | ****** | -0.39 | -0.35 | ***** | -0.46 | -0.35 | ***** | -0.55 | -0.35 | ****** |
| IC1 | 2.6 | 2.54 | * | 2.91 | 2.54 | ******* | 2.92 | 2.54 | ******* | 2.95 | 2.54 | ******* | 3.04 | 2.54 |  |
| BertzCT | 2.71 | 2.5 | *** | 2.88 | 2.51 | ******* | 2.86 | 2.51 | ******* | 2.98 | 2.51 | ******* | 3.06 | 2.51 | ******* |
| J | 1.88 | 2.24 | *** | 1.75 | 2.23 | ******* | 1.76 | 2.22 | ******* | 1.74 | 2.22 | ******* | 1.63 | 2.22 | ******* |
| dchi3 | 3.18 | 2.56 | *** | 4.51 | 2.56 | ******* | 4.17 | 2.58 | ******* | 6.11 | 2.59 | ******* | 6.1 | 2.59 | ******* |
| Chi6ch | 0.15 | 0.1 | *** | 0.17 | 0.1 | ******* | 0.07 | 0.04 | ******* | 0.19 | 0.1 | ******* | 0.22 | 0.1 | ******* |
| S12 | 11.15 | 5.74 | *** | 8.91 | 6.01 | ******* | 9.1 | 6.01 | ******* | 7.14 | 6.06 | **-** | 8.27 | 6.05 | **-** |
| S18 | 0.58 | 0.18 | *** | 1.08 | 0.19 | ******* | 1.18 | 0.19 | ******* | 0.62 | 0.2 | ***** | 0.67 | 0.2 | **-** |
| S21 | 1.19 | 1 | - | 2.34 | 0.98 | ******* | 2.22 | 0.99 | ******* | 3.23 | 1 | ***** | 2.68 | 1 | ***** |
| PEOEVSA0 | 8.75 | 10.7 | *** | 17.75 | 10.45 | ******* | 16.48 | 10.49 | ******* | 25.73 | 10.53 | ******* | 26.42 | 10.55 | ******* |
| MRVSA2 | 2.12 | 4.09 | *** | 5.32 | 3.95 | ***** | 6.11 | 3.94 | ****** | 4.91 | 3.97 | **-** | 0.49 | 3.98 | ******* |
| slogPVSA7 | 6.39 | 2 | *** | 10.01 | 2.11 | ******* | 10.36 | 2.13 | ******* | 11.15 | 2.22 | ****** | 10.21 | 2.23 | ***** |
| EstateVSA9 | 8.34 | 8.43 | - | 15.6 | 8.28 | ******* | 13.42 | 8.34 |  | 25.39 | 8.36 | ******* | 25.42 | 8.38 | ******* |
| bcutp1 | 3.91 | 3.8 | *** | 3.98 | 3.81 | ******* | 3.98 | 3.81 | ******* | 4.01 | 3.81 | ******* | 4.06 | 3.81 | ******* |
| bcutp2 | 3.61 | 3.4 | *** | 3.65 | 3.41 | ******* | 3.64 | 3.41 | ******* | 3.66 | 3.42 | ******* | 3.76 | 3.42 | ******* |
| bcutp9 | 1.98 | 1.92 | *** | 2.06 | 1.92 | ******* | 2.06 | 1.92 | ******* | 2.09 | 1.93 | ******* | 2.1 | 1.93 | ******* |
| bcutp10 | 1.77 | 1.67 | *** | 1.82 | 1.67 | ******* | 1.82 | 1.67 | ******* | 1.84 | 1.67 | ******* | 1.87 | 1.67 | ******* |
| Sfinger17 | 3.34 | 1.97 | *** | 4.01 | 2.01 | ******* | 3.42 | 2.03 | ******* | 6.5 | 2.03 | ******* | 8.2 | 2.03 | ******* |
